# Paracoccidioidomycosis Protective Immunity

**DOI:** 10.3390/jof7020137

**Published:** 2021-02-13

**Authors:** Eva Burger

**Affiliations:** Department of Microbiology and Immunology, Universidade Federal de Alfenas, Alfenas 37130-001, Brazil; eva.burger@unifal-mg.edu.br

**Keywords:** paracoccidioidomycosis, innate immunity, acquired immunity

## Abstract

Protective immunity against *Paracoccidioides* consists of a stepwise activation of numerous effector mechanisms that comprise many cellular and soluble components. At the initial phase of non-specific innate immunity, resistance against *Paracoccidioides* comes from phagocytic polymorphonuclear neutrophils, natural killer (NK) cells and monocytes, supplemented by soluble factors such as cytokines and complement system components. Invariant receptors (Toll-like receptors (TLRs), Dectins) which are present in cells of the immune system, detect patterns present in *Paracoccidioides* (but not in the host) informing the hosts cells that there is an infection in progress, and that the acquired immunity must be activated. The role of components involved in the innate immunity of paracoccidioidomycosis is herein presented. Humoral immunity, represented by specific antibodies which control the fungi in the blood and body fluids, and its role in paracoccidioidomycosis (which was previously considered controversial) is also discussed. The protective mechanisms (involving various components) of cellular immunity are also discussed, covering topics such as: lysis by activated macrophages and cytotoxic T lymphocytes, the participation of lytic products, and the role of cytokines secreted by T helper lymphocytes in increasing the efficiency of *Paracoccidioides*, lysis.

## 1. Immune System Activation and Main Effector Mechanisms That Provide Protection against Microorganisms

The general function of the immune system is to provide protection against the causative agents of infectious diseases and to provide constant surveillance against the emergence and uncontrolled growth of tumoral cells. In most cases, the establishment of the immune response is a positive and desired event—one responsible for providing protection against infections. In fact, if the immune response is insufficient (as it occurs in immunodeficiency diseases) the result is a higher state of susceptibility to all types of infectious diseases.

It should be kept in mind that the immune system has to be maintained under tight control, and if some of these controls are lost then deleterious effects may occur. For example, the immune system is committed to specifically tolerate self-components. This is achieved by two mechanisms; the first is central tolerance, eliminating autoreactive lymphocytes in the primary or central lymphoid organs (bone marrow and thymus), and the second is peripheric tolerance, which is achieved in the secondary lymphoid organs (spleen and lymph nodes), by eliminating or regulating those autoimmune cells that remain. If tolerance is broken, autoimmunity and autoimmune diseases develop. These are some of the situations in which the development of an immune response is deleterious. Other situations of this sort are represented by hypersensitivity reactions which are commonly known as allergies—these situations are encompassed by the term immunopathology.

Concerning infections, the immune response is highly desirable and usually provides sufficient protection to allow a healthy life. This article presents an overall view of how a protective immune response is established, identifying the main cellular and soluble components involved, and how the immune system is activated. The various effector mechanisms involved in the normal immune response mounted against infectious agents are explained, focusing on infections caused by fungi—specifically by *Paracoccidioides brasiliensis*.

Most mycoses occur in individuals with defective immune responses, a population of susceptible individuals that has greatly increased in recent years, due to the important developments in the treatment of cancers and the success of organ transplants. Therefore, as this much-increased number of individuals with compromised immunity lead normal lives, more and more fungal species, erroneously considered to be “avirulent”, have demonstrated that they are quite competent in causing infections by invading and colonizing such hosts. However, there are also thermodimorphic fungi such *Paracoccidioides*, which are pathogenic *per se*; that is, they are able to infect, invade and cause disease in normal hosts which have adequate immune responses. 

An effective immune response against an infecting agent depends on the characteristics of both the microorganism and the host. The effector mechanism(s) amongst the array of the existing immune responses that will be the most efficient against a particular pathogen, depends greatly upon its habitat within the host—i.e., whether it is intracellular (inhabitant of the cytosol or contained in phagosomes), or it is resident in the extracellular spaces. *Paracoccidioides* thrives in all spaces cited above, therefore a combination of all the effector immune mechanisms should be activated in the combat against this fungus. Therefore, the characteristics of protective immunity that may apply to infections caused by *Paracoccidioides* will be briefly discussed.

In extracellular microorganisms, sometimes the initial innate immunity is good enough to control the infection using the complement system (activated through the alternative activation pathway and the lectins pathway), or by natural killer (NK) cells, phagocytic neutrophils and resident monocytes and the triggering of an acute inflammatory reaction. More recently, it was acknowledged that the role of the innate immunity is much more complex than previously considered. 

Some families of invariant receptors, analogous to those originally described in invertebrates (which survive with only the innate immunity), named pattern recognition receptors (PRRs), are able to recognize pathogen-associated molecular patterns (PAMPs), consisting of structures present in microorganisms but not in the mammal hosts. The interaction between PAMPs on the microorganism and PRRs on the immune cells of the host will determine much of the outcome of the infection.

In fungi, PAMPs are components of the fungal cell wall which consequently have an essential role in the survival of the fungus within the host. The wall composition varies between filamentous fungus and yeast, and also between different fungal species and even between strains, but are mainly composed of α and β glucan, chitins, *N*- and *O*-linked mannan. The structure and components of the fungal cell walls, as well as the properties of the diverse PAMPs were the object of numerous studies [1,2,3,4].

The host receptors, known as pathogen recognition receptors (PRRs) have as important representatives in fungal infections, Toll-like receptors (TLR) and Dectin, described, respectively, by Kawai and Akira 2010 [5] and Brown and Gordon 2010 [6]. These receptors, which recognize structures absent from the host cells, are numerous and belong to various families. The main receptors that recognize bacterial cell walls are mostly nucleotide oligomerization domain proteins (NOD), peptidoglycan recognition proteins and *C*-type lectins, whereas Toll like receptors-2 and -4 (TLR-2 and TLR-4) and *C*-type lectins (Dectin-1, Dectin-2, mannose receptor, DC-SIGN) recognize fungal cell wall components. These were the object of excellent reviews [1,2,3,4].

When one of these receptors, which comprise of a whole family of TLR (TLR2, TLR3, TLR4, TLR6, TLR9), Dectin 1 and Dectin 2, Mincle and Dc-Sign, are linked to PAMPS they initiate many intracellular pathways. They also activate nuclear factors and trigger cascades of signals in the cells in which they are present, that results in the activation of the cells and the production (amongst many products) of soluble peptides called cytokines, that will also exert powerful influence on the acquired immune response that will follow. These events occur in fungal infections and were described in detailed by Romani [1]. Cytokines act mostly on nearby cells (paracrine effect) but can act on the cell that produced them (autocrine effect) or even on distant cells reached by the bloodstream (endocrine effect). 

If the infection is not controlled at this stage, the acquired immunity will be activated and specific antibodies will be highly effective, allowing the activation of the complement system by the classical pathway and targeting phagocytosis by specific recognition of fungal antigens. Opsonization by the Fc region of the immunoglobulin IgG isotype and by complement fragment C3b will enhance the effectiveness of phagocytosis. Neutralization of toxins by antibodies is vital, but this is not the case in *Paracoccidioides* infection since none have yet been described. Another mechanism is the antibody-dependent cell cytotoxicity (ADCC), in which cytotoxic cells that have receptors for IgG Fc lyse the fungus by mechanisms involving perforin, granzyme and the induction of apoptosis.

It can be stated that protective immunity against *Paracoccidioides* while in the extracellular space is expected to be essentially humoral. However, *Paracoccidioides* is found intracellularly, ingested by phagocytes (contained in phagosomes) as a typically exogenous antigen. At this stage innate immunity is no longer efficient, acquired humoral immunity is neutralized by the intracellular localization of the fungus, and the acquired cellular immune response is activated.

The main mechanism involved in the cellular immune response is the activation of T helper lymphocytes (Th), which are the central core of the acquired immune response. Th cells are identified (as are all T lymphocytes) by the presence of the T cell receptor (TCR) responsible for the specific recognition of antigens adequately presented. T helper lymphocytes are extremely particular in their recognition of antigens and their subsequent activation. These cells respond only to peptides presented by macrophages, B lymphocytes and dendritic cells which share the functional definition of antigen presenting cells (APCs). These latter cells can be divided into inflammatory and myeloid subpopulations, which lead to the synthesis of different cytokines. The APCs must process the antigens originated from, e.g., *Paracoccidioides*, and present them in the context of histocompatibility antigens which are a family of molecules synthesized and expressed as the result of the instructions by the Major Histocompatibility Complex (MHC) genes. *Paracoccidioides,* being a fungal cell, is contained in the phagosome and is processed as an exogenous antigen and presented in the context of major histocompatibility antigens class II (MHC-II) to helper T lymphocytes, in contrast to viruses, that are free in the cytosol and are processed as endogenous antigens and presented in the context of major histocompatibility antigens class I (MHC-I) to cytotoxic T lymphocytes. 

Once antigen processing and presentation is achieved and the T lymphocyte TCR specifically recognizes the antigen, there is a cell–cell interaction, in which the antigenic peptide is shared by the T lymphocyte TCR and the APC histocompatibility molecule. This interaction is further stabilized by the interactions between the CD4^+^ receptor present on Th lymphocytes with the constant portion of the Class II histocompatibility antigen. Other pairs of ligands enhance and/or inhibit this T lymphocyte activation. Therefore, the presence of such receptors both on the APCs and the T lymphocytes render the proceeding immune response mediated by T lymphocytes more or less efficient. As an example, CD86 or CD80 expressed on APCs provide co-stimulatory signals for T lymphocyte activation when interacting with CD28 present on T cells. Microorganisms may inhibit the expression of these receptors, contributing to their virulence mechanisms. On the other hand, the interaction of the CTLA-4 receptor expressed only transiently on activated cytotoxic (Tc) and helper (Th) lymphocytes but constitutively on regulatory (Treg) lymphocytes with the receptor B7 on APCs, results in inhibition. Therefore, if microorganisms enhance the expression of these latter pair of receptors, then the immune response is inhibited-which is favorable for the pathogen.

When activation of T lymphocytes is achieved, a whole set of metabolic pathways is activated; resulting in the synthesis of cytokines which control the behavior of other cell populations. It should be remembered that different signaling pathways may be initiated by the recognition of different receptors present at the APCs that activate alternative responses. 

At this point, cytokines produced by Th cells activate macrophages, enhancing the metabolism of these cells which in turn produce elevated concentrations of metabolites of the oxygen and nitrogen oxidative pathways. This is necessary as during the innate immune response, the macrophages often ingest the fungi but are unable to proficiently kill them. However, when the macrophages are activated, they are rendered competent enough to control the internalized microorganisms. The subpopulation of Th cells responsible for macrophage activation is the Th1 which can be differentiated from other subpopulations by the set of cytokines produced. These subpopulations of T helper lymphocytes were first described in 1986 by Mosmann et al. [7] and they are not characterized by expressing different receptors, but by producing different patterns of cytokines named Th1 (IL-2, γ IFN) and (IL4, IL-5, IL-6) cytokines.

The paramount role of T helper CD4^+^ lymphocytes in helping B lymphocytes to terminally differentiate into antibody-secreting plasmacytes, and in recruiting and activating other cells from the immune response through the production of various cytokines has already been focused on in this review. Depending on the prevailing pro-inflammatory/anti-inflammatory environment, these Th lymphocytes can differentiate into Th1 or Th2 subsets which produce distinct cytokine patterns and have somewhat different functions. The former are involved in the elimination of intracellular pathogens (fungi in the present context) and the latter are involved in responses against extracellular pathogens. Almost 15 years ago, a third subset of CD4^+^ T cells was identified as Th17. These cells are activated by the secretion of IL-23 by dendritic cells and other APCs after the uptake and processing of pathogens. Th17 cells were found to have a pro-inflammatory role against extracellular bacteria and fungi (but also in the development of autoimmune diseases), both mediated by the secretion of the cytokine IL-17 by these cells. 

In recent years, the Th1/Th2 framework was complemented by the discovery that naive peripheral Th (Th0) cells differentiate into Th1, Th2, Th17, Th9, Th22 and regulatory T cells (Tregs). Regulatory T cells (Tregs) are a population of CD4^+^ T cells which have the unique role in the immune response of controlling excessive inflammation and immoderate immune responses and thus are crucial in suppressing aberrant or excessive pathological immune responses. Tregs are activated through the specific T-cell TCR receptor, but their effector function is nonspecific. They regulate the local inflammatory response through cell-to-cell contact and secretion of cytokines such as IL-9, IL-10, and TGF-ß. There are a number of identified Treg subpopulations: natural Tregs (N), natural naive Tregs (Nn), Th3 cells and regulatory T class 1 (Tr1) cells. New T helper lymphocyte subsets (Th9, Th17, and Th22 cells), and their respective cytokine products (IL-9, IL-17, and IL-22), have been reported to play critical roles in many pathologies—infectious or not [8]. Those studied in *Paracoccidioides* infections are reported below.

Another phenomenon that results from being activated is the enhanced expression of histocompatibility antigens, which are paramount to efficient antigen presentation to T lymphocytes. Activated macrophages are efficient in killing most intracellular microorganisms. However, with some intracellular bacteria such as *Mycobacterium tuberculosis*, and with *Paracoccidioides*, the activation of macrophages by Th1 cytokines is not enough to eliminate the internalized *Paracoccidioides*. In these cases, the cytoplasmic membranes of the macrophages become coalesced, the macrophages undergo differentiation, originating multinucleated giant cells which are highly effective phagocytes. These cells are surrounded by a crown of T lymphocytes that constantly activate them, and contain intact or dead microorganisms. The formation is encircled by fibroblasts which are fibrocytes secreting collagen fibers of various kinds which thus encircles the fungi. These structures are known as granulomas, in which microorganisms and cells of the immune system are in a delicate balance. The fungus is not eliminated, but it is unable to disseminate. If this precarious equilibrium is lost, as often happens in various circumstances leading to immunosuppression (old age, suppressive therapy, infections with immunosuppressive agents such as HIV), the structure of the granulomas is altered, and the contained microorganisms may proliferate and cause acute disease.

Some microorganisms that are contained in phagocytic vacuoles (isolated from the cell components), can escape from this partial segregation and immerse in the cytosol, escaping from the deleterious substances to which they would be exposed within the phagolysosome. Instead, the microorganism remains in direct contact with the cytosol, behaving as a virus. It is known that such microorganisms can be challenged by the cytotoxic T lymphocytes (Tc), which have the characteristic TCR of a T lymphocyte and also a receptor named CD8. This cell population is less proficient in synthesizing cytokines than their T helper counterparts, but they are very efficient effector cells. This means that they are able to kill cells that contain microorganisms such as *Paracoccidioides* or viruses in their cytosol, as well as tumoral cells. The aforementioned Th lymphocytes activate these Tc lymphocytes as they activate macrophages, also by means of secreting cytokines.

Having mentioned that Th lymphocytes are the cells controlling the immune response, their effect on humoral immunity should be discussed. As already mentioned, Th2 lymphocytes also exert influence by secreting cytokines on other target cells (the B lymphocytes), which are the precursors of the antibody-secreting plasmacytes. Therefore, humoral immunity which is represented by soluble antibody molecules (responsible for the protection of the extracellular phase of the microorganisms), is preceded by a complex interaction of various cell populations and subpopulations, and therefore cellular immunity. This cellular immunity is responsible for the protection against intracellular microorganisms and is highly complex, involving different lymphocyte subpopulations and macrophages as well as soluble factors like cytokines. In this context, both cellular and humoral immunity are composed by an intricate cooperation of cells and soluble factors.

One of the fundamental characteristics of the immune response is auto-regulation which means that as the immune response controls the infection, its intensity diminishes and the whole response wanes, thus avoiding excessive and eventually pathological responses. One of the mechanisms involved in self-regulation involves a subpopulation of T cells called regulatory T lymphocytes (Treg, with FoxP3 phenotype), which is one of the T cell subsets mentioned before.

## 2. *Paracoccidioides* and Paracoccidioidomycosis Main Features

Paracoccidioidomycosis is a term that was officially recognized during a meeting of mycologists held in 1971 (Medellin, Colombia), under the auspices of the Pan American Health Organisation. Paracoccidioidomycosis is a systemic mycosis, which can be asymptomatic or manifest as a disease. Paracoccidioidomycosis was first described in 1908, in Brazil, by Adolpho Lutz who reported the presence of the fungus in patients, along with its isolation in culture and pointed out the sporulation differences between it and the fungus *Coccidioides immitis*. After the discovery of the disease that was fatal at that time, a period of attempts at isolation and study of its etiological agent followed. In 1912, the Italian bacteriologist Alfonso Splendore classified the etiologic agent of paracoccidioidomycosis within the genus *Zymonema* and proposed the name *Zymonema brasiliense*. The consensually accepted classification as *Paracoccidioides brasiliensis* (*P. brasiliensis*) was made in 1930 by Floriano de Almeida and persisted until quite recently, when molecular biology techniques allowed us to obtain the now definitive concept that the etiologic agents of paracoccidioidomycosis are the *Paracoccidioides brasiliensis* complex and *Paracoccidioides lutzii*. Taxonomically they are classified in the kingdom Fungi, phylum Eumycota; sub-division Deutromycotina, class Eurotiomycetes, order Onygenales, family Ajellomycetaceae, genus Paracoccidioides. The genus paracoccidioides consists of five species, namely *P. brasiliensis* sensu stricto, *P. americana* or PS2, *P. restrepiensis* or PS3, *P. venezuelensis* or PS4 and *P*. *lutzii*.

Over the next four decades, studies were developed that described skin and mucosal lesions as well as the involvement of lymph nodes, adrenal glands, lungs and bones. It was established that the “entrance door” of the fungus was pulmonary and the histopathology of paracoccidioidomycosis was studied. The existence of a gamut of clinical forms was characterized, as well as asymptomatic infection. The prognosis for patients has changed with the introduction of increasingly efficient treatments: using sulfonamides, antibiotics, imidazole and triazole derivatives, but treatment is still problematic due to the scarce therapeutic choices. The knowledge derived from the studies of the epidemiology and immunology of paracoccidioidomycosis in patients was added to that obtained by the development of experimental models of the disease.

*Paracoccidioides* is a pleomorphic organism, which depends on the temperature for the expression of its morphology. Due to its thermal dimorphism, it can be found in one of two forms: mycelial or yeast-like. The mycelial form constitutes the saprophytic form of the fungus and is observed when cultivated at temperatures from 25 to 27 °C. In this phase, *Paracoccidioides* presents colonies with a cottony, whitish appearance, and microscopically they show the presence of hyaline, thin and septate filaments. The yeast form is the parasitic form of the fungus which can be reproduced in culture from 35 to 37 °C, presenting rounded cells with double walls and single or multiple buds. This multiple exosporulation is the characteristic budding form called “ship rudder wheel” or “Mickey mouse” form. Despite being a eukaryotic cell with a cell wall made up of chitin, the presence of Golgi complex has not been demonstrated. There is still much to study about *Paracoccidioides*, since it is known only in its imperfect form.

Knowledge about the epidemiology of paracoccidioidomycosis is scarcer than that of other mycoses. Paracoccidioidomycosis is most often found in adult males aged 20 to 60 years, living and/or working in rural areas. As agricultural work is performed by both men and women, the disparity of clinical manifestations between the genders (up to 15 times more common in men) cannot be explained by different exposure rates. As there is no compulsory notification of paracoccidioidomycosis, its real incidence is unknown. The largest number of cases are registered in Brazil, Venezuela and Colombia (South America) and in Guatemala (Central America). No paracoccidioidomycosis cases have been found outside a territorial strip between Mexico City to the North and Buenos Aires to the South. The cases diagnosed in other parts of the world correspond to patients who contracted the disease in Latin America and who developed it later. There are as yet no epidemics of this disease. 

The relationship between *Paracoccidioides* and its ecological niche has yet to be discovered, probably due to the long incubation period that elapses between contact with the fungus and the development of the disease. It becomes difficult to establish a cause-effect relationship or even to determine the location of exposure to the fungus. Our ignorance about the “habitat” of *Paracoccidioides* and the small number of acute cases, makes it impossible to study the initial events of paracoccidioidomycosis infection. Paracoccidioidomycosis is a non-contagious disease, with few family cases being reported. It is believed that the infection occurs by inhaling forms of the saprophytic phase of its etiologic agent, *Paracoccidioides*. There is no indication of the participation of any other animal species in the epidemiological chain of paracoccidioidomycosis, despite natural infection having been described in armadillos, but which have no symptomatic disease and are not participants in the cycle. Although the ecological niche of the fungus has not yet been definitively established, there are reports of its isolation from soil in Argentina, Venezuela and Brazil. The most favorable climatic conditions for the growth of *Paracoccidioides* seem to have an average temperature from 17 to 24 °C, moderate humidity and abundant vegetation in regions of tropical and subtropical forest, with the presence of rivers. The absence of paracoccidioidomycosis in extensive locations within endemic regions indicates the need for *Paracoccidioides* for very well-defined niches.

Currently, there is a consensus that the infection of humans by *Paracoccidioides* occurs through inhalation of propagules of the mycelial phase of the fungus (conidia). Once established, lung lesions can remain unnoticed or lead to the development of the disease, with or without dissemination to other organs or systems. The most common outcome of *Paracoccidioides*–human interaction is the development of paracoccidioidomycosis infection. This is defined as an asymptomatic infection, caused by *Paracoccidioides* in normal individuals, who live in an endemic area and are reactive to the skin test with the paracoccidioidin antigenic preparation. From the lungs, *Paracoccidioides* is drained to regional lymph nodes, constituting the primary focus of the paracoccidioidomycotic lesion. Once established, the disease can evolve in different ways. Characteristics of both the host (genetic heritage, sex, age, health status, immune system integrity) and fungus (strain, degree of virulence), as well as human–fungus interaction (number of infectious particles, route of infection) jointly determine the evolution of paracoccidioidomycotic infection. In this aspect, the ability of the female hormone estrogen to bind to *Paracoccidioides* and inhibit its transformation from mycelial to yeast forms may constitute an important protective mechanism for women [9].

The clinical manifestations of paracoccidioidomycosis are diverse, characterized mainly by chronic granulomas, with a high frequency of pulmonary and mucocutaneous lesions. Other organs commonly involved are lymph nodes, adrenals, liver, spleen, intestine, bone marrow and central nervous system. The lungs are clinically involved in more than 80% of cases and lung infection is usually accompanied by injuries to other organs. There have been several attempts to classify the clinical forms of paracoccidioidomycosis, but only recently a consensus was obtained which highlights the fact that the disease has important clinical variations and is difficult to systematize. 

The most diverse criteria were used in the various classification attempts: topography of the lesions, immunological criteria, pathogenesis of the disease, clinical aspects of the patients. Among the proposed classifications, the most accepted is the division of paracoccidioidomycosis into paracoccidioidomycosis infection and paracoccidioidomycosis disease; with the latter being acute or sub-acute (in young people) and chronic (in adults). The chronic form can be sub-divided into mild, moderate and severe. 

In general, two types of evolution of paracoccidioidomycosis can be characterized: (i) rapid evolution—with a tendency of the fungus to spread, undermining the general health conditions of the patient (acute or sub-acute form) and (ii) slow evolution—with smaller granulomatous lesions and fewer organs and systems involved (chronic form). Polar forms of paracoccidioidomycosis can be observed, with a wide range of intermediate forms. The concepts covered here have been thoroughly reviewed by Shikanai-Yasuda et al. [10] and Franco et al. [11].

The laboratorial diagnosis of paracoccidioidomycosis is based on the microscopic findings of fungi with the characteristic morphology of *P. brasiliensis*, on serology employing a variety of techniques as well as an array of antigenic preparations. Of these, the glycoprotein of 43 kDa (GP43) the primary antigen of *Paracoccidioides* and main diagnostic antigen elicits the production of circulating antibodies in most patients [12]. Further studies revealed that the peptide P10, originating from gp43, was a promising vaccinal candidate for a vaccine [13].

Paracoccidioidomycosis is a systemic mycosis with severe clinical consequences. Although *Paracoccidioides* is susceptible to most antifungal drugs, treatment is long and complicated. The antifungal drugs most commonly used to treat PCM patients, are amphotericin B, sulfamidic drugs and azoles [10]. Therefore, it is necessary to search for new therapeutic alternatives that reduce treatment time and, consequently, side effects. Such new broadened approaches were the object of recent reviews [14,15].

## 3. Immune System Activation and Main Effector Mechanisms That Provide Protection against *Paracoccidioides*

The events participating in the interaction of *Paracoccidioides* and the components of the host immune system are discussed below in a temporal fashion, in the approximate order in which cellular and humoral components are called to action in an infection by *Paracoccidioides*. Starting with cell and receptor initiation and soluble factors of the innate immunity, followed by components of the adaptive immunity. It should be kept in mind that many phenomena occur at the same time and that the classification in innate and acquired immunity is merely formal, although very useful to try to apprehend the overall picture that develops. The data has been separated into that which has been obtained using experimental animal models of infection and that which has been collected by analyzing materials from infected patients.

Immunological memory was always considered to be an exclusive and defining feature of the acquired immune system. However, a growing body of literature indicates that activation of the innate immune system can also result in an enhanced responsiveness to subsequent triggers—a property that has been termed “trained immunity”. There are not yet reports in the literature of induction of broad protection against *Paracoccidioides* through innate immune mechanisms previously induced, i.e., the “trained immunity”. 

### 3.1. Immune System Components and Their Activation

#### 3.1.1. Cells of the Innate Immunity

The first cell population that *Paracoccidioides* will encounter will be neutrophils. The role of polymorphonuclear neutrophils in paracoccidioidomycosis is not entirely clarified. Classically it was demonstrated that human peripheric blood neutrophils were able to phagocytose *Paracoccidioides* yeast cells, but not to lyse the ingested yeast cells [16]. 

Difficulties arise from the fact that neutrophils obtained from peripheral blood present different fungicidal activity than those that were collected from the peritoneal cavity after stimulation with irritant agents. In fact, intra-peritoneal neutrophils obtained from mice sensitized with dead *Paracoccidioides* yeast cells were able to lyse the fungus in an in vitro model [17]. Murine neutrophils, obtained by stimulation with *Paracoccidioides* were also shown to be able to lyse yeast cells, and this activity was correlated with the enhanced ability of these cells to generate an oxidative burst [18].

Therefore, these classic studies point out that these cells were effective against *Paracoccidioides*. However, a very important fact was annotated quite early in the research history of this mycosis: *Paracoccidioides* infection somehow renders neutrophils from patients less competent to deal with the initial phases of the infection, as it was found that neutrophils from normal population were more effective than those from patients in terms of both ingesting and destroying the fungus [19].

In an experimental ex vivo model, differences in lytic capacity towards *Paracoccidioides* were found in neutrophils obtained from a resistant mouse strain and from a susceptible strain, indicating that competence of this cell population might be implicated in the overall resistance against the fungus [20]. More recently it was demonstrated that *Paracoccidioides* infection in susceptible mice caused the impairment of normal neutrophil maturation, leading to the premature release from the bone marrow of immature cells. On the other hand, the production and liberation of mature neutrophils was normal in resistant mice. These differences in neutrophil maturation may account for the different efficiency in facing *Paracoccidioides* infection at its onset [21].

Curiously, although neutrophils are the first cells to migrate to the site of the infection, a characteristic feature in paracoccidioidomycosis is the persistence of these cells in the lesions, at later stages of the disease. In fact, neutrophilic infiltrates near *Paracoccidioides* with altered morphology were found in chronic phases in infected mice [22]. As we now understand, neutrophils have a dual role constituting the first line of defense, phagocytosing and killing *Paracoccidioides* by generation of reactive oxygen species during the respiratory burst during the innate immune response. They also modulate the acquired immune response that follows the production of pro- and anti-inflammatory cytokines, and therefore help to mount an efficient adaptive immunity that would lead to protective immunity. 

Another cell population from the innate immune response that *Paracoccidioides* will have to deal are the natural killer (NK) cells. These cells are the cytotoxic cells from the innate immune response and although the recognition mechanisms differ, their lytic mechanisms are remarkably similar to those employed by the cytotoxic T lymphocytes from the adaptive immune response. This is described later in the immune response against *Paracoccidioides* infection, either by directly killing the yeast cells or by recognizing and killing the infected cells. Longhi et al. [23] showed that NK cells are able to recognize and be directly activated by *Paracoccidioides* yeast cells (thus participating actively in the immune response) either by directly killing the fungi or *Paracoccidioides*-infected cells, and also that NK cells from patients with paracoccidioidomycosis present reduced cytotoxic activity.

Another cell population that *Paracoccidioides* encounter early in their journey into the host are the monocytes. It was shown that human monocytes or monocyte-derived macrophages efficiently ingested *Paracoccidioides*, but were unable to control their multiplication (unless stimulated), as will be described later [24]. 

Macrophages are known for their role as effector cells, phagocytosing and lysing ingested microorganisms both in the innate and in the acquired phases of the immune response, and their important role in the protective immunity against *Paracoccidioides* has been thoroughly studied. It has been classically demonstrated that phagocytosed *Paracoccidioides* can multiply inside macrophages unless they are activated by cytokines [25]. It was also shown that the functional blockade of macrophages enhanced the severity of *Paracoccidioides* infection both in susceptible and resistant mouse strains [26]. 

A pathogen first interacts with invariant receptors found mostly in cells from the innate immune response but also present in the lymphocytes, characteristic cells of the acquired immune response. Toll-like receptors (TLR) were the first invariant receptors described and are the best-characterized. The essential role of TLR-2 and TLR-4 in the recognition of *Candida albicans*, *Aspergillus fumigatus* and *Cryptococcus neoformans* antigens was reported [27]. Over the last two decades, the contributions to the literature of TLR interactions with various fungi were extremely rich, and expertly reviewed by some authors which are important contributions to the field [28,29,30].

Although not as numerous as with other fungi, the contributions on the interactions of TLR and *Paracoccidioides* are significant. It was shown that *Paracoccidioides* increased the expression of TLR-2 and decreased that of TLR-4 in human neutrophils, suggesting that the fungus uses TLR4 as a way to gain access to these cells. This however did not kill the *Paracoccidioides*, therefore suggesting a pathogenic role for TLR4 in this context [31]. A decreased expression of TLR2, TLR4 and TLR1 on monocytes and increased expression on neutrophils was also found [32]. The interaction of the immunodominant *Paracoccidioides* antigen gp43 with TLR2 and TLR4 neutrophils from healthy human individuals resulted in an upregulated expression of these receptors, followed by production of the inflammatory cytokine IL-17A and of the eicosanoids PGE2 and LTB4 [33]. This finding is different from that of Balderramas [34] who showed that the release of LTB4 by neutrophils did not involve TLR2 or TLR4. 

Although it was shown that the interaction with TLR-2 and TLR-4 TLR-9 was important during *P. brasiliensis* infection, the effects of this signaling were found to be heterogeneous. In some circumstances, deleterious consequences for the host were reported. In fact, a non-protective role was proposed for TLR2 as a result of increased expression of the gene for TLR-2 that was found after infection in susceptible (but not resistant) mice [35]. In addition, because IL-10 production and a preferential TLR-2 expression were observed in susceptible mice (in contrast to the resistant ones) this suggests that TLR2 facilitates the access of *Paracoccidioides* into macrophages [36]. 

The importance of TLR in various aspects of the interaction with *Paracoccidioides* were further explored by employing animals that had genes for specific TLR knocked-out. The effect of TLR-3 linking was also shown to be deleterious to the host. A negative effect was also reported after binding of *Paracoccidioides* with TRL-3. Cytotoxic T-CD8 lymphocytes were more effective in TLR-3 knock-out mice than in their normal counterparts and the macrophages of these TLR3^−^/^−^ mice also had higher fungicidal activity and NO production than macrophages obtained from TLR-3^+/+^ controls [37]. In a model of experimental immunization, TLR 9^−/−^ mice were found to produce more antibodies than the control mice, mostly of the IgG1 and IgG3 subtypes, demonstrating that TLR-9 plays a role in the protection induced by immunization with rPb27 and confirming the importance of TLR-9 in the initial protection against paracoccidioidomycosis [38]. In fact, Menino et al. [39] showed that TLR9 has a protective role early after intravenous infection with *P. brasiliensis*, as more infected TLR9^−/−^mice died during the first 48 h post infection than wild type mice did. They also demonstrated that TLR-9 recognizes *P. brasiliensis* yeasts form as well as its DNA.

After *Paracoccidioides* is sensed by TLR-2 and TLR-4, the signaling adaptor molecule myeloid differentiation primary response protein 88 (MyD88) molecule is involved, and through this pathway the activation of macrophages is triggered and inflammatory cytokines are produced. It was shown that mice lacking this signaling adaptor were more susceptible to *Paracoccidioides* infection and were unable to mount protective immunity to control fungal growth and dissemination and to develop organized granulomas, which led to shorter survival time than their MyD8^+/+^ controls [40].

Dectin-1 is a C-type lectin-like cell surface receptor which is present in neutrophils, macrophages and other cells of the immune system that binds specifically to *β*-1,3 glucans present on the fungal cell wall. This is therefore fundamental to the induction of innate and adaptive immunity following fungal recognition, by enhancing phagocytosis by an increase in the respiratory burst and production of reactive oxygen species through the production of inflammatory cytokines.

Various groups have reported the participation of Dectin-1 in the recognition of *Paracoccidioides*, either alone [41,42] or together with TLR [32,43,44]. This recognition was shown to trigger many events, some realized in the activation phase of the immune response and others related to the effector phase of the immune response. In the former case, the production or enhanced production of cytokines such as IL-12, IL-10, PGE2 and LTB4 by human neutrophils has been described by Balderramas et al. [34] and TNF-α, IL-1*β*, IL-18, IL-12, IL-8, IL-17 and IL-10 as reported by Romagnolo et al. [45] both using material from patients. In the latter case, it can be cited that the release of extracellular traps (NETs) and consequent extracellular killing of the fungus [42] and a direct correlation between expression of Dectin-1 and *Paracoccidioides* lysis indicating a positive role in the fungal killing by monocytes and neutrophils [46]. 

Altogether these results strongly suggest the participation of TLRs and Dectin in *Paracoccidioides* linkage, internalization and the consequent activation of the immune response against the fungus. Many aspects of the innate immune response against *Paracoccidioides* have been the object of an expert revision by Calich et al. [47].

It is known that Dectin-1 binds to any β-1,3-glucan-containing fungal species. To avoid the interaction with immune cells, fungi have developed hiding PAMPs such as β-1,3-glucan and chitin with different molecules. For instance, a mannan layer in these fungi works as a protective shield that prevents interaction of β-1,3-glucan with immune receptors [48]. However, the recent findings by Yadav et al. [49]. point out that mannan does not shield the fungus from recognition by macrophages, although it does shield it from monocytes; this points to the fact that the shielding by mannan can be protective for the fungi against some, but not all cells of the immune system.

#### 3.1.2. Cells from the Interface of Innate and Acquired Immunity

Macrophages are highly effective cells in in their role of effector cells, phagocytosing and destroying internalized *Paracoccidioides*. Moreover, they also play a determinant role in the establishment of an efficient immune response against this fungus, acting at the interface of the innate and acquired immune responses (as APCs) as shown by the data below.

Macrophages obtained from mice resistant to *Paracoccidioides* infection express Class II histocompatibility antigens persistently, whereas macrophages from susceptible mice express it only transiently [50]. This well-known phenomenon is regulated by different cytokines, with IL-4 inducing an ephemeral expression and IFN-γ a sustained expression. Susceptible and resistant mouse strains (although not exactly characterizing a Th1/Th2 dichotomy) produce a quite different cytokine milieu throughout *P. brasiliensis* infection, predominating type 2 in the former and type 1 cytokines in the latter mice. Therefore, the cytokine production pattern can influence the efficacy of *Paracoccidioides* antigen presentation to Th cells and the consequent establishment of a protective acquired immune response. More recently, an increased expression on APCs of Class II histocompatibility antigens and of the CD86 receptor was observed in the lungs of *Paracoccidioides*-infected mice [51]. 

Employing the same model but focusing on another cell population functionally classified as an APC (the dendritic cells), Ferreira et al. [36] observed that dendritic cells from resistant mice were more phagocytic than cells from susceptible mice, in parallel to distinct cytokine secretion patterns. They found low concentrations of IL-10, IL-12, and TNF-α for DCs in resistant mice and high concentrations of TNF-α and IL-10 in susceptible mice suggesting that susceptibility to *Paracoccidioides* infection could result from the involvement of regulatory dendritic cells. Other groups have reported different cytokine pattern production; namely, a balanced production of pro- and anti-inflammatory cytokines in the resistant mice and predominance of pro-inflammatory cytokines synthesis by the susceptible mice, elicited by the induction of different dendritic cell subpopulations in these mouse strains by *Paracoccidioides* infection. The resistant mice developed both myeloid and plasmacytoid dendritic cells whereas susceptible mice produced preferentially pro-inflammatory myeloid dendritic cells, and these subpopulations caused expansion of effector and regulatory T cells in the resistant mice suppression of T lymphocyte responses in the susceptible mice. The expansion of these two different dendritic cells subpopulations resulted in an effective T lymphocyte response in the resistant mice and in an impaired proliferation of effector T cells in the susceptible mice, eventually by activating different Th subpopulations through different adaptor molecules [52]. The in vitro differentiation of human monocyte-derived dendritic-cell subpopulations is influenced by the presence of *Paracoccidioides* cell wall fractions such as the alkali-insoluble β-glucan-rich fraction and the alkali-soluble α-glucan-rich fraction. The α-glucan fraction stimulates the differentiation of a dendritic-cell subpopulation that, through the secretion of cytokines, can negatively affect polarization towards Th1 immune response and play a role enhancing fungal pathogenesis [53]. The existence of different dendritic cell subpopulations and their various activation pathways have already been mentioned as well as the different cytokines and T cell subpopulations that these APCs will elicit. Notch signaling has an important role in DC maturation and has been shown to contribute effectively to the maturation of dendritic cells, as well as to the activation of the T lymphocyte response in *Paracoccidioides* infections mediated by this cell population [54]. 

Optimization of antigen presentation by dendritic cells was employed in experimental models aiming for vaccination. Magalhães et al. [55] showed that dendritic cells primed with a *Paracoccidioides* component (P10) (derived from the immunodominant gp43 antigen) stimulated a Th1 cytokine response and reduced the numbers of fungi in the lungs. This was also achieved with immunocompromised mice in a disease model that simulated the most aggressive form of paracoccidioidomycosis [56]. In this study, the authors isolated and generated monocyte differentiated dendritic cells from infected mice, pulsed them with P10 and then used them in the therapy of PCM in syngeneic mice [57].

#### 3.1.3. Cells of the Acquired Immunity

Acquired immune response is dominated by T and B lymphocytes, cells that have the unique property of specifically recognizing antigens. Absence of either one of these cell populations renders a *Paracoccidioides* infection much more severe. In athymic (nude) mice which are unable to provide an environment favorable for T lymphocyte maturation, the course of the disease was more severe, resulting in a higher mortality than that observed in control mice [58]. A higher mortality rate was also verified in mice with the absence of B Lymphocytes, caused by the knockout of the respective gene [59].

Paracoccidioidomycosis is characterized by presenting different clinical forms of complicated classification. The standing concept is that these depend more on the immune response of the hosts than on the virulence variants of the fungus. The severe form of the disease, with involvement of various organs and rapid evolution to death are accompanied by the gradual loss of specific cellular immune responses and high titer of specific antibodies. In comparison, mild forms of the disease present few localized lesions, leading to healing and are parallel to maintained cellular immune responses [60,61] and low levels of specific antibodies [62].

After the discovery of Th1 and Th2 T lymphocytes subpopulations, this concept was reviewed, and it was agreed that a totally polarized Th1/Th2 pattern of immune response does not develop. A good prognosis in patients and resistant behavior in murine models correlates with a preferential secretion of type-1 cytokines, whereas a bad prognosis in patients [63,64] and susceptible behavior in the mouse model [65,66] is associated with a preferential type-2 cytokine production. The subsequent discovery of new T Lymphocyte subpopulations and cytokines and the research on their role in *Paracoccidioides* infection has improved the comprehension of the cytokine network in paracoccidioidomycosis. The Th1/Th2 paradigm solved the mystery of why high titers of antibodies were not protective in this disease: Th2 cytokines, preferentially produced in the severe forms of the disease are fundamental for B lymphocytes proliferation and differentiation into antibody producing plasmacytes.

Ever since the participation of cellular immunity was demonstrated to be essential for the establishment of a protective immunity against *Paracoccidioides* infection, research was intense and the knowledge on the role of many T lymphocytes subpopulations increased considerably. On the other hand, the role of B lymphocytes, plasmacytes and their products (antibodies) in immunoprotection was, for many years, a subject involved in controversy.

The participation of T lymphocyte subpopulations has been studied in experimental models and the important role of helper T lymphocytes of T CD4^+^ phenotype was demonstrated. It was shown that in susceptible mice these cells were either absent or anergic, but in resistant or intermediately resistant mice this cell population was present and actively involved in effective delayed-type hypersensitivity reaction resulting in protection [67]. The role of cytotoxic T lymphocytes with TCD8^+^ phenotype in protective immunity against *Paracoccidioides* was also demonstrated, showing that this cell population expands during infection [68] and is responsible for the control of the fungal load in mice of any genetic background, as demonstrated by the increased severity of the infection in mice depleted of these cells [69].

The participation of cytotoxic T lymphocytes was also studied in patients. A lower frequency of cytotoxic T lymphocytes was detected in *Paracoccidioides* patients than that found in individuals with strong cellular response against the fungus (presenting *Paracoccidioides* infection). The overall results strongly indicated that cytotoxic T lymphocytes obtained from *Paracoccidioides* patients are poorly activated and produce basal levels of cytotoxic granules, resulting in defective cytotoxic activity and consequently, a low capacity to kill the fungus [70].

The activation or inhibition of lymphocytes results from the sequential interactions of pairs of receptors, as already mentioned in this manuscript. The latter event was reported by Campanelli et al. [71] who recovered more CTLA-4^+^ T lymphocytes from the blood and from tissue lesions obtained from *Paracoccidioides* patients than those found in healthy controls, as well as higher levels of CTLA-4 expression, thus indicating that *Paracoccidioides* is regulated at both the local and systemic levels. 

However, Lozano et al. [72] when studying the CTLA-4 gene polymorphisms that have been correlated with disfunctions of the immune response, associated it with susceptibility or resistance to *Paracoccidioides*. Another pair of receptors previously mentioned (CD28 and CD86) have been studied and found to be diminished in paracoccidioidomycosis patients as compared with heathy controls [73]. Although single nucleotide polymorphisms (SNPS) represent minor genetic differences between individuals, they can nevertheless impact mRNA splicing, nucleocytoplasmic export, and mRNA stability and translation. When present within a coding sequence, they can lead to amino acid substitutions that modify the activity of the protein.

Mendonça et al. [74] analyzed two polymorphisms in the cytokine IL-4 gene of 76 paracoccidioidomycosis patients and of 73 controls from an endemic area. One of the polymorphisms was located in the promoter region and the second in intron-3 of the IL-4 gene. They found significant correlation between the intron-3 polymorphism and the occurrence of disease. They found that one genotype (RP2/RP2) was associated with infection by *Paracoccidioides* and another (RP1/RP1) with resistance to the fungus. The authors discuss that their results are similar to those obtained by Bozzi et al. 2006, [75] who, analyzing IL-10 and TNF-α gene polymorphisms in paracoccidioidomycosis, demonstrated an association between the IL-10 polymorphism and disease. The role of regulatory T cells (Tregs) on protective immunity in fungal infections is controversial, but in some cases (such as sporotrichosis) it was recently shown that Tregs have a deleterious role, as depletion of this cell population improves the protective antifungal immunity [76]. In paracoccidioidomycosis, the role of Tregs seems to be negative, since adverse effects of this cell subpopulation were reported by various authors reporting data from experimental models and from patients.

Analyzing the effect of early depletion of the Treg cell population in a murine model, it was found that the absence of these cells resulted in less severe tissue pathology and abolished the enhanced mortality observed in susceptible mice. This demonstrates that Treg lymphocytes are deleterious to both the progressive and regressive forms of paracoccidioidomycosis [77]. The same group showed that Treg cell depletion is also protective in ongoing paracoccidioidomycosis, as it rescues infected hosts from progressive and potentially fatal PCM [78]. Peron et al. [51] detected increased numbers of Tregs in *Paracoccidioides*-infected mice in parallel with higher levels of IL-10 originating both from APCs and cytotoxic T lymphocytes as already cited. Their results suggested that *Paracoccidioides* could modulate the immune response, influencing the balance between regulatory Tregs and Th17 polarization. 

This immunomodulation was also studied in *Paracoccidioides* patients. Treg lymphocytes were found to be present in the lesions and peripheral blood from patients with the chronic form of paracoccidioidomycosis, suggesting local and systemic regulation of the immune response in this mycosis [79]. Ferreira et al. [80] reported that the patients with active disease had more Treg cells than the patients who received treatment (or controls) and proposed that the immunosuppression observed was due to Treg cells acting either directly by cell–cell interaction, or as an effect of the suppressive cytokines produced. Genaro et al. [81] verified that dendritic cells pulsed with *Paracoccidioides*-induced Tr1 lymphocytes in vitro. The authors also detected higher numbers of Treg1 lymphocytes (a regulatory T lymphocyte subset which secrete IFN-γ and IL-10) in patients presenting severe clinical forms of the disease (acute and severe chronic forms) compared to those in patients with mild clinical forms (chronic form) and proposed that Treg1 lymphocytes could be involved in the deficient immune response developed against *Paracoccidioides* which would cause the severe clinical forms of this mycosis. The role of Treg in *Paracoccidioides* infection was expertly reviewed quite recently by Calich et al. [82], but recently new contributions on the subject have been made. 

The enzyme indoleamine 2,3 dioxygenase-1 (IDO1) induces Treg cells and inhibits Th17 differentiation which therefore influence the outcome of many infectious processes, including those caused by *Paracoccidioides*. The absence of this enzyme resulted in an increase and decrease, respectively, in Th1 and Treg cells at the infection site; higher *Paracoccidioides* loads and tissue pathology resulting in increased mortality rates [83].

The direct effect of *Paracoccidioides* infection in hampering the protective immune response that the host could mount against was also the object of previous studies. *Paracoccidioides* infection can hinder the development of a protective immune response by invading primary lymphoid organs. *Paracoccidioides* was found in the thymus and in the bone marrow. In the former, acute infection promotes thymic alterations leading to a defective repertoire of peripheral T cells [84]. In the latter, it causes major alterations in the maturation of many cell populations including neutrophils during hematopoiesis and also of inducing an inflammatory profile, by the expression of IL-6, IL-17, TNF-α, and TGF-β cytokines [21]. 

### 3.2. Immune System Effector Mechanisms 

#### 3.2.1. Role of Hormones and Cytokines

*Paracoccidioides* infection in humans occurs through inhalation of conidia, present in the mycelial phase of the fungus, followed by mycelium to yeast conversion, which is depressed only in women, due to the effect of steroid hormones, as described in the classical papers of Restrepo et al. [85] and of Salazar et al. [86]. Following this, Stover et al. [87] described the existence of a thermal labile high-affinity estrogen-binding site and a low-affinity binding site in the yeast form of *Paracoccidioides* as well as an estrogen-binding site in the mycelium phase of the fungus and demonstrated in vitro that estradiol inhibited the morphological transition of *Paracoccidioides* from the mycelial form to the yeast form. The authors proposed that estradiol either blocks or delays the morphological transition from the mycelial form into the yeast form of *Paracoccidioides* observed in tissues, acting as a protective factor for women in reproductive age and explaining the lower frequency of clinical disease in women as compared with men.

It is quite difficult to didactically separate cells belonging to the innate immunity or to the acquired immunity. It is even more difficult to try to separate cytokines that are mediators of the innate immunity from those that are mediators of the adaptive immunity. The difficulty in locating each cytokine in one of these two compartments of the immune response is due to some of the inherent characteristics of these factors. In addition to exerting their influence in nearby or distant cell populations, cytokines have other particular properties. They are pleiotropic, meaning that one cytokine may have two cell populations as target cells, therefore one can be more involved in the innate immune response and the other in the acquired immunity. Additionally, different cell populations can produce the same cytokines. The cytokine network permeates all aspects of the immune response and as a consequence, it is extremely complicated to tag one cytokine as characteristic of one arm or one phase of the immune response. Therefore, the actions of a cytokine or a group of cytokines in an approximate order of their participation in the events during a paracoccidioidomycosis infection are presented.

The activation of phagocytic cells such as neutrophils [88,89], monocytes [90,91], monocytes/macrophages [24], pulmonary macrophages [92], peritoneal macrophages [93] by individual cytokines such as TNF-α and IFN-γ and the ulterior lysis of *Paracoccidioides* has been extensively demonstrated, as well as the effectiveness of a mixture of proinflammatory cytokines [94]. 

Interleukin 15 (IL-15) is produced principally by monocytes and macrophages during the innate immune response. When this cytokine was incubated with human monocytes and challenged with *Paracoccidioides* it induced significantly higher levels of TNF-α, IL-10 and IL-15 and also enhanced fungicidal activity [95]. When it was incubated with neutrophils, IL-15 also augmented the ability of this cell population to kill *Paracoccidioides* [96].

The stimulation by IL-12, IL-15 or IL-18 cytokines of human neutrophils challenged with *Paracoccidioides* resulted in production of IFN-γ which was much increased when IL-12 and IL-15 were employed [97].

Using ELISA, the analysis of the concentration of mRNA cytokines expression in supernatant produced by monocytes from healthy subjects challenged with *Paracoccidioides* detected the production of IL-1, IL-6, IL-10 and TNF-α, but the levels of IL-1, IL-6, and IL-10 were higher when a virulent strain was employed. This early and continuous production of pro- and anti-inflammatory cytokines during infection created an imbalance in the cytokine microenvironment which the authors suggest leads to unhampered fungal growth [98].

Further contributions attested the participation of various cytokines in experimental and human paracoccidioidomycosis. The protective role of IFN-γ [99] as well as the detrimental role of IL-4 [100] in the outcome of experimental infection with *Paracoccidioides* was also demonstrated.

A systemic influence of cytokines in the overall outcome of *Paracoccidioides* infection was demonstrated by the important publications which confirmed that the cytokine milieu is markedly different throughout controlled disease and in progressive disease, both in human paracoccidioidomycosis and in experimental models. In fact, secretion of cytokines TNF-α and IFN-γ tended to be higher when the host controlled the infection and that of IL-4 and TGF- β when the infection remained uncontrolled [101]. Both IFN-γ and IL-4 increased in patients, but secretion of IFN-γ tended to increase while that of IL-4-secretion decreased with the treatment [102,103]. In fact, polymorphisms in IL-10 (-1082 G/A) and TNF-α (-308 G/A) cytokines genes have been correlated with PCM. It was found that the IL-10-1082G allele, when homozygous, could be associated with an increased risk of contracting the illness [75]. No such correlation was found for the polymorphism in TNF-α, which was found in higher levels in the serum of PCM patients [101]. A negative role of some cytokines in the outcome of *Paracoccidioides* infection was also shown. The macrophages of mice with their gene for IL-10 knocked-out, had higher production of pro-inflammatory cytokines and were more efficient in phagocytosing and lysing *Paracoccidioides*. Early development of efficient T cell responses and an effective control fungal load without excessive tissue pathology were found in these mice, resulting in lower mortality rates [104]. The participation of IL-6 was also evaluated. Pre-treatment of human monocytes with IL-6 and challenged with *Paracoccidioides* caused an increase in IL-6 (autocrine loop) and a decrease in TNF-α production, rendering the monocytes more permissive for fungal growth [105].

The role of the IL-1 cytokines family was also studied. The IL-1 family includes cytokines, seven ligands with agonist activity (IL-1α, IL-1β, IL-18, IL-33, IL-36α, IL-36β, IL-36γ) and four members with antagonistic activities (IL-1Ra, IL-36Ra, IL-37, IL-38). One cytokine of this group, the IL-1β, has a unique property of being produced by an intracellular multimolecular complex called inflammasome that is activated by pathogens such as *Paracoccidioides* or by damage signs produced by the host. When phagocytes are exposed to microorganisms such as *Paracoccidioides* they synthesize IL-1β in its inactive form, which is activated through enzymatic pathways similar to those of the complement system with the participation of the inflammasome (a cytosolic multiprotein complex).

Resistance to fungal infection was associated with inflammasome activation and active IL-1 β production. It was demonstrated that a *Paracoccidioides* infection resulted in inflammasome activation and production of IL-1β via the NLRP3 inflammasome [106]. It was also shown that Nlrp3-dependent inflammasomes contribute to host protection against *P. brasiliensis* by a mechanism involving caspase-1 activation and an IL-18-dependent proinflammatory response. This was confirmed by increased susceptibility and permissiveness for fungal growth of knockout mice for Nlrp3, caspase-1 and IL-18, as shown by an augmented fungus burden in IL-18 knockout mice [107]. Resistance to *Paracoccidioides* was also demonstrated to be associated with activation of inflammasome together with Dectin-1 expression [41].

The same correlation of cytokines of the IL-1 family with resistance to *Paracoccidioides* was observed in patients. Higher intracellular protein expression of NLRP3, CASP-1, ASC, and IL-1β and increased priming in mRNA levels of NLRP3 inflammasome genes in monocytes isolated from patients with a chronic form of paracoccidioidomycosis was also reported in comparison to controls [108]. De Castro et al. [109] showed that NLRP3 inflammasome activation and cytokines production by DCs are dependent on a series of events such as ROS generation, endosome acidification, and K^+^ efflux and involve *Paracoccidioides* recognition by dectin-1 and Syk phosphorylation. They also reported that NLRP3 inflammasome is essential for the activation/expansion of Th1/Th17 cells and its inhibition leads to an increased frequency of Th2 and Treg cells and proposed that it is essential for the induction of the initial inflammatory response and in the development of the acquired immune response associated with resistance to infection.

More recently, newly discovered cytokines of the Il-1 family were added to the cytokine context that characterizes different clinical forms of paracoccidioidomycosis [110]. Polymorphisms related to IL12, IL18 and IFN were investigated in paracoccidioidomycosis patients separated in terms of their clinical presentations in acute form (AF), multifocal chronic (MC), or unifocal chronic (UC) forms of PCM and non-infected controls. They observed an increased risk to develop severe forms of the disease (AF and MC) in individuals with IL-18 polymorphisms. However, also, a protective role of IL-18 polymorphism was possibly linked to higher levels of IL-18 at different periods of the course of the disease. No association of clinical forms and IL12 and/or IFN polymorphisms were found [111].

The Th17 cell population has an important role in anti-infective protective immunity, including mycoses. The production of Th17-associated cytokines (IL-6, IL-23, and IL-17A) during experimental paracoccidioidomycosis was analyzed. The production of all Th17-associated cytokines increased in the lung and that of IL-17A increased in vitro, suggesting that Th17-associated cytokines are involved in the modulation of the immune response to *Paracoccidioides* [37]. Gardizan et al. [33] reported that gp43-stimulated human PMNs produced IL-17. This cytokine was also produced by human monocytes from healthy individuals IL-17A after incubation with *Paracoccidioides* via activation of the Dectin-1 receptor [45].

The role of Tregs has already been addressed in this manuscript. Here we will focus on the anti-inflammatory role of IL-27 (a cytokine of the IL-12 family) which is secreted by APCs that induce Treg cells to inhibit Th1 and Th17 responses by the effect of the cytokine IL-10. Higher serum concentrations of IL-27 were found in paracoccidioidomycosis patients presenting the most severe and disseminated forms of this mycosis (acute form and severe chronic form) as compared to patients with mild chronic form. In the lesions of the patients, IL-27 was found associated with both dendritic cells and macrophages. The data suggest that IL-27 may have a role in the deficient immune response to *Paracoccidioides* that leads to severe and disseminated forms of the disease [81].

#### 3.2.2. Role of Antibodies

Infection with *Paracoccidioides* elicits the production of specific antibodies, but only recently were these shown to have protective properties. Before that, antibodies were employed as reliable diagnostic tools. Even when monoclonal antibodies were developed, their immediate application was serological follow-up of patients or infected animals. The contributions that led to the recent concept of protective humoral immunity will be herein presented.

The importance of B cells in resistance to *Paracoccidioides* was shown by a higher mortality rate, numbers of viable *Paracoccidioides* in the lungs, and larger granulomatous lesions observed in B Lymphocyte-B cell-knockout mice. This deficiency was reverted by passive transfer of immune serum from their control counterparts [59].

The pioneer works that showed protective activity of the humoral compartment of the adaptive immune response were done employing passive immunization with monoclonal antibodies directed against major *Paracoccidioides* antigens. Two different monoclonal antibodies directed against a 70 kD glycoprotein from *Paracoccidioides* conferred protection, as observed by the reduced numbers of viable fungi and granulomatous lesion sizes found in the lungs of experimentally infected mice [112].

Passive immunization with some monoclonal antibodies of IgG2a e IgG2b isotypes against Gp 43 (which is the major *Paracoccidioides* antigen employed for diagnostic tests) resulted in enhanced IFN-γ levels in the lungs and increased fungal phagocytosis leading to higher nitric oxide production by macrophages and reduced fungal burden and decreased pulmonary inflammation [113]. Later, it was shown that this major *Paracoccidioides* epitope contains the internal peptide P10 that elicits an IFN-γ-mediated protective Th1 immune response [114].

A strong in vitro inhibitory activity of monoclonal antibodies against a *Paracoccidioides* glycolipid antigen was demonstrated by Toledo et al. [115]. More recently, another MAb that recognizes a secreted 75-kDa protein with phosphatase activity inhibited *Paracoccidioides* growth in vitro and reduced lung CFU in vivo was reported by Xander [116]. Working with *Paracoccidioides lutzii*, Thomaz et al. were able to induce protective immunity with monoclonal antibodies to heat shock protein 60 and induce a protective immune response [117].

Polyclonal antibodies to *Paracoccidioides* components were also shown to be protective, opsonizing the fungi and as a consequence increasing phagocytosis in a murine intratracheal infection model [118] and causing reduction in the fungal burden and formation of well-organized granulomas [119].

Very recently, it was shown that Dectin-1 engagement could prime IL-18 expression and protective antibodies generation in vivo, suggesting a link between Dectin-1 mediated innate immune response and adaptive humoral immunity [120].

#### 3.2.3. Lysis within Phagocytes and Granulomas

One of the many consequences of cytokines on cells is their activation, which on many occasions is accompanied by an increased production of products that are deleterious to *Paracoccidioides*.

In classical studies conducted by McEwen et al. [121], high lethality was obtained for *Paracoccidioides* isolates differing in virulence employing a combination of non-lethal concentrations of H_2_O_2_ and KI, independently of the virulence isolate. When lysis was performed by activated macrophages, more active compounds were responsible for the *Paracoccidioides* killing: nonoxidative mechanisms [122], and the combination of H_2_O_2_ and NO [93].

Nitric oxide (NO), a nitrogen intermediate produced by an oxidative mechanism involving the catabolism of l-arginine by the enzymatic action of inducible nitric oxide synthase (iNOS) is one of the major microbicidal products of macrophages that are active against intracellular microorganisms such as fungi. However, its role in paracoccidioidomycosis is far from clarified. A protective role of NO at the initial phases of an experimental *P. brasiliensis* infection (but not at later stages) was demonstrated [123]. Later, NO was shown to modulate the chronic inflammatory response induced by *Paracoccidioides* leading to tissue degradation, and decreasing extracellular matrix synthesis by controlling inflammatory cytokines and matrix metalloprotein compounds that provide enzymatic activity. The negative effects of NO, observed at the later phases of *Paracoccidioides* infections, could be associated with loose granulomas and high fungal dissemination and result in the progression of the disease [124]. The overall results seem to indicate that absence of NO leads to a better outcome of experimental paracoccidioidomycosis. These findings were in concordance to those of Bernardino et al. [125], who reported that NO production by mice deficient in inducible nitric oxide synthase (iNOS) resulted in enhanced synthesis of TNF-α, less severe disease and increased survival times of the infected mice. However, Livonesi et al. [126] who were analyzing the role of iNOS concluded that it contributed to resistance against *Paracoccidioides* infection. They suggested that because mice deficient in this enzyme were unable to produce nitric oxide, to form organized granulomas or to control *Paracoccidioides* multiplication and therefore died a short time after infection having produced high levels of Th1 (IL-12, IFN-γ and TNF-α) and Th2 (IL-4 and IL-10) cytokines. In addition, Nishikaku et al. [124] found necrotic granulomas in iNOS-deficient mice; suggesting an important role for this enzyme in the genesis of organized compact granulomas which are effective for containment of *Paracoccidioides*.

As seen before in this text, granulomas constitute an active defense mechanism against microorganisms and the ability to mount and maintain organized granulomas is quintessential for *Paracoccidioides* dissemination control. The role of cellular immune response in this phenomenon was shown by the development of expansive lesions with low collagen deposits and increased fungal load in athymic mice in contrast with the presence of compact granulomas with intense deposits of collagen fibers and control of fungal dissemination, observed in their euthymic controls [127,128].

In the controlled infection of mice resistant to *Paracoccidioides*, the granulomas were either well-defined and encapsulated (with predominance of type I collagen) or had residual characteristics with sparse collagen deposits. The fungi found in these granulomas were either massively destroyed near neutrophils or degenerated within pseudo xantomatous macrophages, indicating control of the infection in both cases. On the other hand, the granulomas found in susceptible mice were small, numerous (with less tendency for encapsulation) and with the presence of type III collagen fibers [22]. The protective role of granulomas was confirmed by Souto et al. [129], who demonstrated that TNF-α-deficient mice were unable to mount organized granulomas which led to *Paracoccidioides* dissemination and mortality. A possible influence in fungal dissemination could be due to matrix metalloproteases, particularly of matrix metalloprotease-9. Factors with gelatinolytic activity that were found in granulomas of *P. brasiliensis*-infected mice probably synthesized in multi-nucleated giant cells, macrophages, and lymphocytes present at the lesions.

The compact, encapsulated, or residual granulomas developed by resistant mice had more IFN-γ-positive cells with lymphocyte morphology than the loose and multifocal granulomas of susceptible mice. The presence of this proinflammatory cytokine IFN-γ in resistant mice suggests its effects in activating the macrophages and contributing to the control of fungal dissemination [130]. The presence of the regulatory cytokine TGF-ß was quite different in the granulomas of resistant and susceptible mice. In the former, scarce TGF-ß was found around fibrotic and necrotic areas containing lysed fungi, and in the latter the presence of TGF-ß was marked and could be found on macrophages, giant cells, lymphocytes and fibroblasts (in areas of fibrosis and necrosis) and dispersed in the amorphous extracellular matrix [131]. This suggests that this regulatory cytokine influenced the lesions towards increasing in size and number during the infection [119].

The above papers have shown the presence of cytokines in situ in the lesions developed after *Paracoccidioides* infection. However, cytokines were also implicated in the formation of granulomas. The role of GM-CSF, IFN-γ, TNF-α, IL-10 and TGF-β in the generation of multinucleated giant cells, that are characteristically present in granulomas was studied, stimulating human monocytes with *Paracoccidioides* antigen in an in vitro setting. The authors reported that foreign body-type giant cells were preferentially induced over Langhans-type and detected an important role for GM-CSF in such giant cell formation and enhancement of fungicidal activity [132].

The Th17 lymphocyte subpopulation needs cytokines IL-1, IL-6, and TGF-β for its differentiation and IL-23 for its maintenance. Studies using *Paracoccidiodes* experimental infection showed that deficiency of IL-6, IL-23 cytokines or of the IL-17 receptor IL-17RA impaired the formation of compact granuloma and therefore rendered the animals more susceptible [133].

## 4. Concluding Remarks

The hallmark of paracoccidioidomycosis is the complex spectrum of clinical manifestations, a fact that renders diagnosis and treatment extremely complicated, as expertly reviewed by Shikanai et al. [10]. According to the *International Colloquium on Paracoccidioidomycosis* [11], there are two outcomes of the interaction between humans and *Paracoccidioides*:

(i). Paracoccidioidomycosis infection—without any clinical findings and the infection is diagnosed by a positive delayed type of hypersensitivity reaction to fungal antigens.
(ii). Paracoccidioidomycosis disease—when clinical alterations are found in the residual form in which sequelae are observed after treatment.

*Paracoccidioides* disease is classified in either acute/subacute juvenile form (subdivided in moderate or severe forms) or in chronic adult form (classified in mild, moderate, or severe forms). The severest forms of this mycosis are the acute/subacute forms, in which *Paracoccidioides* disseminates to many organs and there are multiple symptoms reflecting a severe condition and bad prognosis. A prominent finding is a marked eosinophilia. In chronic paracoccidioidomycosis, lungs are the main target organ (although others are involved) and a major laboratory finding is high antibody titers. As discussed in this review, this gamut of clinical forms is due more to the characteristics of the host response than to variability of the infecting *Paracoccidioides* strains, although differences in fungal virulence factors must not be excluded.

Throughout the investigation into the immune response to *Paracoccidioides* infection, there has always been an effort to correlate the clinical manifestations observed in patients as well as the outcome of experimental infections in animal models that mimic the disease with the patterns of the immune response. The study of this mycosis within the framework of the most recent progresses in immunology is the merit of researchers working in the field of immunity to *Paracoccidioides* infection. Thus, always providing a modern concept into which components of the immune system had a positive or a negative participation in the effective combat (or even immunopathogenic manifestations) of the disease. The various explanations to the different clinical manifestations of *Paracoccidioides* infection were always based on the most reliable information and the most modern concepts of immunology.

Therefore, the initial studies conducted almost 50 years ago correctly observed that preserved cellular immunity and production of low antibody titers led to the control of *Paracoccidioides* infection; in contrast with the severe forms of paracoccidioidomycosis observed in the context of elevated levels of specific antibodies and impaired cellular immunity. 

Following this, when the Th1/Th2 lymphocytes and their cytokine production patterns were characterized, the contributions of many researchers allowed the proposal that a pure Th1 response with secretion of cytokines IFN-γ and TNF-α led to *Paracoccidioides* infection. A partial downmodulation of the Th1 response pattern led to mild or moderate chronic forms of human disease and to resistance in mouse models. A polarization towards a Th-2 response pattern resulted in the acute/subacute juvenile forms and in the severe chronic adult form in patients as well as in resistant mice. These responses were characterized, respectively, by total or partial containment of the fungus in compact granulomas, and on the other hand by fungal dissemination from disorganized granulomas. Controlled disease was accompanied by the presence of activated macrophages, numerous neutrophils and Tc lymphocytes, preserved delayed type hypersensitivity reactions, IFN-γ, TNF-α, IL-2 and other Th1 cytokines, and low levels of specific antibodies. Severe disease was characterized by numerous eosinophils, diminished delayed type hypersensitivity reactions, IL-4, IL-5, IL-10 and TGF-β and other Th2 cytokines and high levels of specific antibodies, mainly of IgG4 and IgE isotypes. 

With the inclusion of the more complex set of T cell subpopulations and their cytokines, the present concepts are as follows: a pure Th1 response is still considered to be responsible for *Paracoccidioides* infection, with the presence of IFN γ and IL-2. Mild or moderate chronic forms of paracoccidioidomycosis are now believed to occur in the context of Th17/Th22 lymphocytes and a mixture of cytokines IL-17 and IL-22 (high levels), IFN-γ, TNF-α, IL-2 and IL-10 and IL-4 (variable levels) and with high levels of IgG1 antibodies. The severe acute/subacute juvenile form is considered to occur within the context of a mixed Th2/Th9 response, which results in high levels of cytokines IL-4, IL-5, IL-9, IL-10, TGF-β, and IL-27; low levels of IFN-γ and TNF-α; and high levels of antibodies of the IgG4 and IgE isotypes. 

Therefore, the present concept is that a Th2/Th9 response is responsible for susceptibility to paracoccidioidomycosis, and that a Th1 and Th17/Th22 response is responsible for resistance against *Paracoccidioides*. However, the high levels of IL-12 and IL-17can be protective against *Paracoccidioides* infection, but can also be deleterious by causing excessive inflammatory response and fibrosis. 

The overall conclusion about protective immunity against *Paracoccidioides* is that the fungus activates an extremely complex set of mechanisms. The host answers with an array of responses, comprehending cells and soluble factors of the innate and the acquired immune response. 

Whenever the knowledge on subject of immunity in paracoccidioidomycosis has been pursued, the researchers involved have tried to compose the picture of the immune mechanism involved in the development of either a protective, effective immunity or of an inadequate response that allows progressive disease. Both in animal models and in patients, this quest has been elusive as there are nuances of control of the disease that reflect in the many clinical forms. However, it can be said with certainty that the pioneer researchers were correct, and an intact and efficient cellular immunity is paramount to reach a cure or a controlled disease. In more detail, protective immune response against *Paracoccidioides* is conducted by activator inflammatory cytokines, activated effector cells and a measure of control of excessive inflammation through regulatory cell populations and cytokines. This author is certain that over the course of the next decade, future research will lead to the discovery of the participation of new (as yet unknown) sets of lymphocytes and cytokines in the protective immune response against *Paracoccidioides* infection.

## References

[1] Romani L. (2011). Immunity to fungal infections. Nat. Rev. Immunol..

[2] Dambuza I.M., Gordon D Brown G.D. (2015). C-type lectins in immunity: Recent developments. Curr. Opin. Immunol..

[3] Netea M., Brown G., Kullberg B., Gow N.A.R. (2008). An integrated model of the recognition of *Candida albicans* by the innate immune system. Nat. Rev. Microbiol..

[4] Sukhithasri V., Nisha N., Biswas L., Kumar A., Biswas R. (2013). Innate immune recognition of microbial cell wall components and microbial strategies to evade such recognitions. Microbiol. Res..

[5] Kawai T., Akira S. (2010). The role of pattern-recognition receptors in innate immunity: Update on Toll-like receptors. Nat. Immunol..

[6] Brown G.D., Gordon S. (2001). A new receptor for β-glucans. Nature.

[7] Mosmann T.R., Cherwinski H., Bond M.W., Giedlin M.A., Coffman R.L. (1986). Two types of murine helper T cell clone. I. Definition according to profiles of lymphokine activities and secreted proteins. J. Immunol..

[8] Schmitt N., Ueno H. (2015). Regulation of human helper T cell subset differentiation by cytokines. Curr. Opin. Immunol..

[9] Shankar J., Restrepo A., Clemons K.V., Stevens D.A. (2011). Hormones and the resistance of women to paracoccidioidomycosis. Clin. Microbiol. Rev..

[10] Shikanai-Yasuda M.A., Mendes R.P., Colombo A.L., Queiroz-Telles F., Kono A.S.G., Paniago A.M.M., Nathan A., Valle A.C.F.D., Bagagli E., Benard G. (2017). Brazilian guidelines for the clinical management of paracoccidioidomycosis. Rev. Soc. Bras. Med. Trop..

[11] Franco M., Montenegro M.R., Mendes R.P., Marques S.A., Dillon N.L., Mota N.G.S. (1987). Paracoccidioidomycosis: A recently proposed classification of its clinical forms. Rev. Soc. Bras. Med. Trop..

[12] Camargo Z.P. (2008). Serology of paracoccidioidomycosis. Mycopathologia.

[13] Travassos L.R., Taborda C.P. (2017). Linear epitopes of *Paracoccidioides brasiliensis* and other fungal agents of human systemic mycoses as vaccine candidates. Front. Immunol..

[14] Santos L.A., Grisolia J.C., Malaquias L.C.C., Paula F.B.A., Dias A.L.T., Burger E. (2020). Medication association and immunomodulation: An approach in fungal diseases and in particular in the treatment of paracoccidioidomycosis. Acta Trop..

[15] do Carmo Silva L., de Oliveira A.A., de Souza D.R., Barbosa K.L.B., Freitas E., Silva K.S., Carvalho Júnior M.A.B., Rocha O.B., Lima R.M., Santos T.G. (2020). Overview of antifungal drugs against paracoccidioidomycosis: How do we start, where are we, and where are we going?. J. Fungi.

[16] Goihman-Yahr M., Rothenberg A., Bretaña A., Istúriz G., Rosquete R., Avila-Millán E., Viloria N., Saavedra de Borges N., Carrasquero M., Pérez de Fernández B. (1989). Digestion of killed *Paracoccidioides brasiliensis* by neutrophils. Mycopathologia.

[17] McEwen J.G., Brummer E., Stevens D.A., Restrepo A. (1987). Effect of murine polymorphonuclear leukocytes on the yeast from of *P. brasiliensis*. Am. J. Trop. Med. Hyg..

[18] Brummer E., Sugar A.M., Stevens D.A. (1985). Enhanced oxidative burst in immunologically activated but not elicited polymorphonuclear leukocytes correlates with fungicidal activity. Infect. Immun..

[19] Kurita N., Oarada M., Ito E., Miyaji M. (1999). Antifungal activity of human polymorphonuclear leucocytes against yeast cells of *Paracoccidioides brasiliensis*. Med. Mycol..

[20] Meloni-Bruneri L.H., Campa A., Abdalla D.P., Calich V.L.G., Burger E. (1996). Neutrophil oxidative metabolism and killing of *P. brasiliensis* after air pouch infection of susceptible and resistant mice. J. Leukoc. Biol..

[21] Sperandio F.F., Bruneri L.H.M., Fernandes G.P., Mendes A.C.S.C., Bani G.M.A.C., Calich V.L.G., Burger E. (2015). Resistance to experimental infection of inbred mice is associated with an efficient neutrophil mobilization and activation by mediators of inflammation. Resistance to *P. brasiliensis* experimental infection of inbred mice is associated with an efficient neutrophil mobilization and activation by mediators of inflammation. Mediat. Inflamm..

[22] Xidieh C.F., Lenzi H.L., Calich V.L.G., Burger E. (1999). Influence of the genetic background on the pattern of lesions developed by resistant and susceptible mice infected with *Paracoccidioides brasiliensis*. Med. Microbiol. Immunol..

[23] Longhi L.N.A., da Silva R.M., Fornazim M.C., Spago M.C., de Oliveira R.T., Nowill A.E., Blotta M.H.S.L., Mamoni R.L. (2012). Phenotypic and Functional Characterization of NK Cells in Human Immune Response against the Dimorphic Fungus *Paracoccidioides* brasiliensis. J. Immunol..

[24] Moscardi-Bacchi M., Brummer E., Stevens D.A. (1994). Support of *Paracoccidioides brasiliensis* multiplication by human monocytes or macrophages: Inhibition by activated phagocytes. J. Med. Microbiol..

[25] Brummer E., Hanson L.H., Restrepo A., Stevens D.A. (1989). Intracellular multiplication of *P.brasiliensis* in macrophages and restriction of multiplication by activated macrophages. Infect. Immun..

[26] Kashino S.S., Singer-Vermes L.M., Burger E., Franco M., Moscardi-Bacchi M., Russo M., Vaz C.A.C., Calich V.L.G. (1995). Effect of macrophage blockade on resistance mechanisms of inbred mice to *Paracoccidioides brasiliensis* infection. Mycopathologia.

[27] Netea M.G., Van der Graaf C., Van der Meer J.W., Kullberg B.J. (2004). Recognition of fungal pathogens by Toll-like receptors. Eur. J. Clin. Microbiol. Infect. Dis..

[28] Netea M.G., Ferwerda G., Van der Graaf C.A., Van der Meer J.W., Kullberg B.J. (2006). Recognition of fungal pathogens by toll-like receptors. Curr. Pharm. Des..

[29] Salazar F., Brown G.D. (2018). Antifungal Innate Immunity: A perspective from the last 10 Years. J. Innate. Immun..

[30] Hatinguais R., Willment J.A., Brown G.D. (2020). PAMPs of the fungal cell wall and mammalian PRRs. Curr. Top. Microbiol. Immunol..

[31] Acorci-Valério M.J., Bordon-Graciani A.P., Dias-Melicio L.A., de Assis Golim M., Nakaira-Takahagi E., de Campos Soares A.M. (2010). Role of TLR2 and TLR4 in human neutrophil functions against *Paracoccidioides brasiliensis*. Scand. J. Immunol..

[32] Bonfim C.V., Mamoni R.L., Blotta M.H. (2009). TLR-2, TLR-4 and dectin-1 expression in human monocytes and neutrophils stimulated by *Paracoccidioides* brasiliensis. Med. Mycol..

[33] Gardizani T.P., Della Coletta A.M., Romagnoli G.G., Puccia R., Serezani A., de Campos Soares A., Dias-Melicio L.A. (2019). 43 kDa Glycoprotein (gp43) from *Paracoccidioides* brasiliensis Induced IL-17A and PGE2 Production by Human Polymorphonuclear Neutrophils: Involvement of TLR2 and TLR4. J. Immunol. Res..

[34] Balderramas H.A., Penitenti M., Rodrigues D.R., Bachiega T.F., Fernandes R.K., Ikoma M.R.V., Dias-Melicio L.A., Oliveira S.L. (2014). Human neutrophils produce IL-12, IL-10, PGE2 and LTB4 in response to *Paracoccidioides brasiliensis*. Involvement of TLR2, mannose receptor and dectin-1. Cytokine.

[35] Loures F.V., Pina A., Felonato M., Calich V.L.G. (2009). TLR2 is a negative regulator of Th17 cells and tissue pathology in a pulmonary model of fungal infection. J. Immunol..

[36] Ferreira K.S., Bastos K.R., Russo M., Almeida S.R. (2007). Interaction between *Paracoccidioides* brasiliensis and pulmonary dendritic cells induces interleukin-10 production and toll-like receptor-2 expression: Possible mechanisms of susceptibility. J. Infect. Dis..

[37] Jannuzzi G.P., de Almeida J.R.F., Amarante-Mendes G.P., Romera L.M.D., Kaihami G.H., Vasconcelos J.R., Ferreira C.P., de Almeida S.R., Ferreira K.S. (2019). TLR3 is a negative regulator of immune responses against *Paracoccidioides brasiliensis*. Front. Cell Infect. Microbiol..

[38] Morais E.A., Chame D.F., Melo E.M., Oliveira J.A.C., Paula A.C.C., Peixoto A.C., Santos L.S., Gomes D.A., Russo R.C., Goes A.M. (2016). TLR 9 involvement in early protection induced by immunization with rPb27 against paracoccidioidomycosis. Microbes Infect..

[39] Menino J.F., Saraiva M., Gomes-Alves A.G., Lobo-Silva D., Sturme M., Rezende J.G., Saraiva M., Goldman G.H., Cunha C., Carvalho A. (2013). TLR9 Activation Dampens the Early Inflammatory Response to *Paracoccidioides brasiliensis*, impacting host survival. PLoS Negl. Trop. Dis..

[40] Loures F.V., Pina A., Felonato M., Feriotti C., de Araújo E.F., Calich V.L.G. (2011). MyD88 signaling is required for efficient innate and adaptive immune responses to *Paracoccidioides* brasiliensis infection. Infect. Immun..

[41] Feriotti C., Bazan S.B., Loures F.V., Araujo E., Costa T.A., Calich V.L.G. (2015). Expression of dectin-1 and enhanced activation of NALP3 inflammasome are associated with resistance to paracoccidioidomycosis. Front. Microbiol..

[42] Bachiega T.F., Dias-Melicio L.A., Fernandes R.K., de Almeida Balderramas H., Rodrigues D.R., Ximenes V.F., de Campos Soares Â.M.V. (2016). Participation of dectin-1 receptor on NETs release against Paracoccidioides brasiliensis: Role on extracellular killing. Immunobiology.

[43] Loures F.V., Araúlo E.F., Feriotti C., Bazan S.B., Calich V.L.G. (2015). TLR-4 cooperates with Dectin-1 and mannose receptor to expand Th17 and Tc17 cells induced by *Paracoccidioides brasiliensis* stimulated dendritic cells. Front. Microbiol..

[44] Rodriguez-Echeverri C., Puerta-Arias J.D., González Á. (2020). *Paracoccidioides* brasiliensis activates mesenchymal stem cells through TLR2, TLR4, and Dectin-1. Med. Mycol..

[45] Romagnolo A.G., de Quaglia E., Silva J.C., Della Coletta A.M., Gardizani T.P., Martins A.T.L., Romagnoli G.G., Kaneno R., Soares A.M.V.C., De Faveri J. (2018). Role of Dectin-1 receptor on cytokine production by human monocytes challenged with Paracoccidioides brasiliensis. Mycoses.

[46] de Quaglia Silva J.C., Della Coletta A.M., Gardizani T.P., Romagnoli G.G., Kaneno R., Dias-Melicio L.A. (2019). Involvement of the Dectin-1 receptor upon the effector mechanisms of human phagocytic cells against *Paracoccidioides brasiliensis*. J. Immunol. Res..

[47] Calich V.L.G., Da Costa T.A., Felonato M., Arruda C., Bernardino S., Loures F.V., Ribeiro L.R.R., Valente-Ferreira R.D.C., Pina A. (2008). Innate immunity to *Paracoccidioides brasiliensis* infection. Mycopathologia.

[48] Hernández-Chávez M.J., Pérez-García L.A., Niño-Vega G.A., Mora-Montes H.M. (2020). Fungal strategies to evade the host immune recognition. J. Fungi..

[49] Yadav B., Mora-Montes H.M., Wagener J., Cunningham I., West L., Haynes K., Brown A.J.P., Gow N.A.R. (2020). Differences in fungal immune recognition by monocytes and macrophages: N-mannan can be a shield or activator of immune recognition. Cell Surf..

[50] Calich V.L.G., Fazioli R.A., Teixeira H.C., Russo M., Singer-Vermes L.M., Burger E., Vaz C.A.C., Torres-Rodrigue J.M. Mechanisms of host-resistance to Paracocidioides brasiliensis. Proceedings of the X Congress of the International Society for Human and Animal Mycoses.

[51] Peron G., Oliveira J., Thomaz L.L., Bonfanti A.P., Thomé R., Rapôso C., Verinaud L.M.C. (2020). *Paracoccidioides* brasiliensis infection increases regulatory T cell counts in female C57BL/6 mice infected via two distinct routes. Immunobiology.

[52] Pina A., Araujo E.F., Felonato M., Loures F.V., Feriotti C., Bernardino S., Barbuto J.A.M., Calich V.L.G. (2013). Myeloid dendritic cells (DCs) of mice susceptible to paracoccidioidomycosis suppress T Cell responses whereas myeloid and plasmacytoid DCs from resistant mice induce effector and regulatory T cells. Infect. Immun..

[53] Souza A.C.O., Favali C., Soares N.C., Tavares N.M., Jerônimo M.S., Veloso P.H., Marina C.L., Santos C., Brodskyn C., Bocca A.L. (2019). New role of *P. brasiliensis* α-glucan: Differentiation of non-conventional dendritic cells. Front. Microbiol..

[54] Jannuzzi G.P., de Almeida J.R.F., Dos Santos S.S., de Almeida S.R., Ferreira K.S. (2018). Notch signaling is required for dendritic cell maturation and T cell expansion in paracoccidioidomycosis. Mycopathologia.

[55] Magalhães A., Ferreira K.S., Almeida S.R., Nosanchuk J.D., Travassos L.R., Taborda C.P. (2012). Prophylactic and therapeutic vaccination using dendritic cells primed with peptide 10 derived from the 43-kilodalton glycoprotein of *Paracoccidioides brasiliensis*. Clin. Vaccine Immunol..

[56] Silva L.B.R., Dias L.S., Rittner G.M.G., Muñoz J.E., Souza A.C.O., Nosanchuk J.D., Travassos L.R., Taborda C.P. (2017). Dendritic cells primed with *Paracoccidioides brasiliensis* Peptide P10 are therapeutic in immunosuppressed mice with Paracoccidioidomycosis. Front. Microbiol..

[57] Silva L.B.R., Taira C.L., Dias L.S., Souza A.C.O., Nosanchuk J.D., Travassos L.R., Taborda C.P. (2019). Experimental therapy of paracoccidioidomycosis using P10-primed monocyte-derived dendritic cells isolated from infected mice. Front. Microbiol..

[58] Burger E., Miyaji M., Sano A., Calich V.L.G., Nishimura K., Miyaji M. (1996). *Paracoccidioides brasiliensis* infection in nude mice: Studies with isolates differing in virulence and definition of their T-dependent and T-independent components. Am. J. Trop. Med. Hyg..

[59] Tristão F.S.M., Panagio L.A., Rocha F.A., Cavassani K.A., Moreira A.P., Rossi M.A., Silva J.S. (2013). B Cell-deficient mice display enhanced susceptibility to *Paracoccidioides brasiliensis* infection. Mycopathologia.

[60] Musatti C.C., Rezkallah M.T., Mendes E., Mendes N.F. (1976). In vivo and in vitro evaluation of cell-mediated immunity in patients with paracoccidioidomycosis. Cell Immunol..

[61] Benard G., Mendes-Giannini M.J., Juvenale M., Miranda E.T., Duarte A.J. (1997). Immunosuppression in paracoccidioidomycosis: T cell hyperresponsiveness to two *Paracoccidioides brasiliensis* glycoproteins that elicit strong humoral immune response. J. Infect. Dis..

[62] Biagioni L., Souza M.J., Chamma L.G., Mendes R.P., Marques S.A., Mota N.G.S., Franco M.F. (1984). Serology of paracoccidioidomycosis. II. Correlation between class-specific antibodies and clinical forms of the disease. Trans. R. Soc. Trop. Med. Hyg..

[63] Benard G., Romano C.C., Cacere C.R., Juvenale M., Mendes-Giannini M.J.S., Duarte A.J. (2001). Imbalance of il-2, IFN-γ and IL-10 secretion in the immunosuppression associated with human paracoccidioidomycosis. Cytokine.

[64] Oliveira S.J., Mamoni R.L., Musatti C.C., Papaiordanou P.M., Blotta M.H. (2002). Cytokines and lymphocyte proliferation in juvenile and adult forms of paracoccidioidomycosis: Comparison with infected and non-infected controls. Microbes Infect..

[65] Calich V.L.G., Vaz C.A.C., Burger E. (1998). Immunity to *Paracoccidioides brasiliensis* infection. Res. Immunol..

[66] Kashino S.S., Fazioli R.A., Cafalli-Favatti C., Meloni-Bruneri L.H., Vaz C.A.C., Burger E., Singer L.M., Calich V.L.G. (2000). Resistance to *Paracoccidioides brasiliensis* infection is linked to a preferential Th1 immune response, whereas susceptibility is associated with absence of IFN-γ production. J. Interferon Cytokine Res..

[67] Chiarella A.P., Arruda C., Pina A., Costa T.A., Valente-Ferreira R.C., Calich V.L.G. (2007). The relative importance of CD4^+^ and CD8^+^ T cells in immunity to pulmonary paracoccidioidomycosis. Microbes Infect..

[68] Loures F.V., Araújo E.F., Feriotti C., Bazan S.B., Costa T.A., Brown G.D., Calich V.L.G. (2014). Dectin-1 induces M1 macrophages and prominent expansion of cd8^+^il-17+ cells in pulmonary paracoccidioidomycosis. J. Infect. Dis..

[69] Cano L.E., Singer-Vermes L.M., Mengel J.A., Xidieh C.F., Arruda C., Aandré D.C., Vaz C.A.C., Burger E., Calich V.L.G. (2000). Depletion of CD8^+^ T cells in vivo impairs host defense of mice resistant and susceptible to pulmonary paracoccidioidomycosis. Infect. Immun..

[70] Burlandy-Soares L.C., Mamoni R.L., Lyra L., Schreiber A.Z., Blotta M.H. (2010). Expression of activation and cytotoxic molecules by peripheral blood lymphocytes of patients with paracoccidioidomycosis. Med. Mycol..

[71] Campanelli A.P., Martins G.A., Souto J.T., Pereira M.S., Livonesi M.C., Martinez R., Silva J.S. (2003). Fas-Fas ligand (CD95-CD95L) and cytotoxic T lymphocyte antigen-4 engagement mediate T cell unresponsiveness in patients with paracoccidioidomycosis. J. Infect. Dis..

[72] Lozano V.F., Lins T.C., Teixeira M.M., Vieira R.G., Blotta M.H., Goes A.M., Silva I.C., Pereira R.W., Bocca A.L., Felipe M.S. (2011). Polymorphism analysis of the CTLA-4 gene in paracoccidioidomycosis patients. Mem. Inst. Oswaldo Cruz..

[73] Bozzi A., Reis B.S., Prado F.L., Pedroso E.P., Leite M.F., Goes A.M. (2004). Modulation of CD28 and CD86 expression in patients with paracoccidioidomycosis in different periods of treatment. Scand. J. Immunol..

[74] Mendonça M.S., Peraçolli M.T.S., Silva-Vergara M.L., Ribeiro S.C., Oliveira R.F., Mendes R.P., Rodrigues V. (2015). High interleukin-4 expression and interleukin-4 gene polymorphisms are associated with susceptibility to human paracoccidioidomycosis. Mem Inst. Oswaldo. Cruz..

[75] Bozzi A., Pereira P.P., Reis B.S., Goulart M.I., Pereira M.C., Pedroso E.P., Leite M.F., Goes A.M. (2006). Interleukin-10 and tumour necrosis factor-α single nucleotide gene polymorphism frequency in paracoccidioidomycosis. Hum. Immunol..

[76] Batista-Duharte A., Téllez-Martínez D., De Andrade C.R., Polesi M.C., Portuondo D.L., Carlos I.Z. (2020). Transient Foxp3(+) regulatory T-cell depletion enhances protective Th1/Th17 immune response in murine sporotrichosis caused by *Sporothrix schenckii*. Immunobiology.

[77] Felonato M., Pina A., de Araujo E.F., Loures F.V., Bazan S.B., Feriotti C., Calich V.L.G. (2012). Anti-CD25 treatment depletes Treg cells and decreases disease severity in in susceptible and resistant mice infected with *Paracoccidioides brasiliensis*. PLoS ONE.

[78] Galdino N.A.L., Loures F.V., de Araújo E.F., da Costa T.A., Preite N.W., Calich V.L.G. (2018). Depletion of regulatory T cells in ongoing paracoccidioidomycosis rescues protective e Th1/Th17 immunity and prevents fatal disease outcome. Sci. Rep..

[79] Cavassani K.A., Campanelli A.P., Moreira A.P., Vancim J.O., Vitali L.H., Mamede R.C., Martinez R., Silva J.S. (2006). Systemic and local characterization of regulatory T cells in a chronic fungal infection in humans. J. Immunol..

[80] de Ferreira Oliveira R.D., da Silva R.M., Blotta M.H.S.L., Mamoni R.L. (2010). Involvement of Regulatory T Cells in the Immunosuppression Characteristic of Patients with Paracoccidioidomycosis. Infect. Immun..

[81] Genaro L.M., Coser L.O., Justo-Junior A.D.S., de Castro L.F., Barreto A.K.F., Rizzato A.E., Trabasso P., Mamoni R.L., Pereira R.M., Cintra M.L. (2020). Association between IL-27 and Tr1 cells in severe form of paracoccidioidomycosis. Cytokine.

[82] Calich V.L.G., Mamoni R.L., Loures F.V. (2019). Regulatory T cells in paracoccidioidomycosis. Virulence.

[83] De Araújo E.F., Feriotti C., Galdino N.A.L., Preite N.W., Calich V.L.G., Loures F.V. (2017). The IDO–AhR axis controls Th17/Treg immunity in a pulmonary model of fungal infection. Front. Immunol..

[84] Di Gangi R., Da Costa T.A., Thomé R., Peron G., Burger E., Verinaud L. (2016). Paracoccidioides brasiliensis infection promotes thymic disarrangement and premature egress of mature lymphocytes expressing prohibitive TCRs. BMC Infect. Dis..

[85] Restrepo A., Salazar M.E., Cano L.E., Stover E.P., Feldman D., Stevens D.A. (1984). Estrogens inhibits mycelium-to-yeast transformation in the fungus *P. brasiliensis*: Implications for resistance of females to paracoccidioidomycosis. Infect. Immun..

[86] Salazar M.E., Restrepo A., Stevens D.A. (1988). Inhibition by estrogens of conidium-to-yeast conversion in the fungus *P. brasiliensis*. Infect. Immun..

[87] Stover E.P., Schar G., Clemons K.V., Stevens D.A., Feldman D. (1986). Estradiol-binding proteins from mycelial and yeast-form cultures of *Paracoccidioides brasiliensis*. Infect. Immun..

[88] Kurita N., Oarada M., Miyaji M., Ito E. (2000). Effect of cytokines on antifungal activity of human polymorphonuclear leucocytes against yeast cells of *Paracoccidioides* brasiliensis. Med. Mycol..

[89] Rodrigues D.R., Dias-Melicio L.A., Calvi S.A., Peraçoli M.T.S., Soares A.M.V.C. (2007). *Paracoccidioides brasiliensis* killing by IFN-γ, TNF-α and GM-CSF activated human neutrophils: Role for oxygen metabolites. Med. Mycol..

[90] Calvi S.A., Peraçoli M.T.S., Mendes R.P., Marcondes-Machado J., Fecchio D., Marques S.A., Soares A.M.V.C. (2003). Effect of cytokines on the in vitro fungicidal activity of monocytes from paracoccidioidomycosis patients. Microb. Infect..

[91] Carmo J.P.M., Dias-Melicio L.A., Calvi S.A., Peraçoli M.T.S., Soares A.M.V.C. (2006). TNF-α activates human monocytes for *Paracoccidioides brasiliensis* killing by an H2O2-dependent mechanism. Med. Mycol..

[92] Cano L.E., Arango R., Salazar M.E., Brummer E., Stevens D.A., Restrepo A. (1992). Killing of *Paracoccidioides brasiliensis* conidia by pulmonary macrophages and the effect of cytokines. J. Med. Vet. Mycol..

[93] Moreira A.P., Dias-Melicio L.A., Peraçoli M.T.S., Soares A.M.V.S. (2008). Killing of *Paracoccidioides brasiliensis* yeast cells by IFN-γ and TNF-α activated murine peritoneal macrophages: Evidence of H2O2 and NO effector mechanisms. Mycopathologia.

[94] Gonzalez A., Sahaza J., Ortiz B., Restrepo A., Cano L. (2003). Production of pro-inflammatory cytokines during the early stages of experimental *Paracoccidioides brasiliensis* infection. Med. Mycol..

[95] Bannwart C.F., Martins R.A., Nakaira-Takahashi E., Dias-Melício L.A., Soares A.M., Peraçoli M.T. (2010). Interleukin-15 augments oxidative metabolism and fungicidal activity of human monocytes against *Paracoccidioides* brasiliensis. Mem Inst. Oswaldo Cruz..

[96] Tavian E.G., Dias-Melicio L.A., Acorci M.J., Graciani A.P., Peraçoli M.T., Soares A.M. (2008). Interleukin-15 increases Paracoccidioides brasiliensis killing by human neutrophils. Cytokine.

[97] Rodrigues D.R., Fernandes R.K., Balderramas H.A., Penitenti M., Bachiega T.F., Calvi S.A., Dias-Melicio L.A., Ikoma M.R., Soares A.M.V. (2014). Interferon-gamma production by human neutrophils upon stimulation by IL-12, IL-15 and IL-18 and challenge with Paracoccidioides brasiliensis. Cytokine..

[98] Kurokawa C.S., Araujo J.P. (2007). Pro- and anti-inflammatory cytokines produced by human monocytes challenged in vitro with *Paracoccidioides brasiliensis*. Microbiol. Immunol..

[99] Cano L.E., Kashino S.S., Arruda C., André D., Xidieh C.F., Singer-Vermes L.M., Vaz C.A.V., Burger E., Calich V.L.G. (1998). Protective role of γ interferon in experimental pulmonary paracoccidioidomycosis. Infect. Immun..

[100] Pina A., Valente-Ferreira R.C., Molinari-Madlum E.E., Vaz C.A.C., Keller A.C., Calich V.L.G. (2004). Absence of interleukin-4 determines less severe pulmonary paracoccidioidomycosis associated with impaired Th2 response. Infect. Immun..

[101] Silva C.L., Figueiredo F. (1991). Tumor necrosis factor in paracoccidioidomycosis patients. J. Infect. Dis.

[102] de Castro L.F., Ferreira M.C., da Silva R.M., Blotta M.H.S.L., Longhi L.N., Mamoni R.L. (2013). Characterization of the immune response in human paracoccidioidomycosis. J. Infect..

[103] Mamoni R., Blotta M.H.S.L. (2006). Flow-cytometric analysis of cytokine production in human paracoccidioidomycosis. Cytokine.

[104] Costa T.A., Bazan S.B., Feriotti C., Araujo E.F., Bassi E.J., Loures F.V., Calich V.L.G. (2013). In pulmonary paracoccidioidomycosis IL-10 deficiency leads to increased immunity and regressive infection without enhancing tissue pathology. PLoS Negl. Trop. Dis..

[105] Siqueira K.Z., Campos Soares A.M., Dias-Melicio L.A., Calvi S.A., Peraçoli M.T. (2009). Interleukin-6 treatment enhances human monocyte permissiveness for Paracoccidioides brasiliensis growth by modulating cytokine production. Med. Mycol..

[106] Tavares A.H., Magalhães K.G., Almeida R.N., Correa R., Burgel P.H. (2013). NLRP3 inflammasome activation by *Paracoccidioides brasiliensis*. PLoS Negl. Trop. Dis..

[107] Ketelut-Carneiro N., Silva G.K., Rocha F.A., Milanezi C.M., Cavalcanti-Neto F.F., Zamboni D.S., Silva J.S. (2015). IL-18 Triggered by the Nlrp3 inflammasome induces host innate resistance in a pulmonary model of fungal infection. J. Immunol..

[108] Amorim B.C., Pereira-Latini A.C., Golim M.A., Ruiz Júnior R.L., Yoo H.H.B., Arruda M.S.P., Tavares A.H., Cavalcante R.S., Mendes R.P., Pontillo A. (2020). Enhanced expression of NLRP3 inflammasome components by monocytes of patients with pulmonary paracoccidioidomycosis is associated with smoking and intracellular hypoxemia. Microbes Infect..

[109] De Castro L.F., Longhi L.N.A., Paião M.R., Justo A.S., de Jesus M.B., Blotta M.H.S.L., Mamoni R.L. (2018). NLRP3 inflammasome is involved in the recognition of *Paracoccidioides* brasiliensis by human dendritic cells and in the induction of Th17 cells. J. Infect..

[110] Alves A.B.R.P., David M.A., de Castro L.F., Silva R.M., Longhi L.N.A., Blotta M.H.S.L., Mamoni R.L. (2017). Differential production of interleukin-1 family cytokines (IL-1β, IL-18, IL-33 and IL-37) in patients with paracoccidioidomycosis: Correlation with clinical form and antifungal therapy. Med. Mycol..

[111] Sato P.K., Busser F.D., Carvalho F.M.C., dos Santos A.G., Sadahiro Aya A.S., Lima D.C., Kono A., Gonçalves S., Moretti M.L., do Carmo L.O. (2020). Polymorphism in the Promoter Region of the IL18 Gene and the Association with Severity on Paracoccidioidomycosis. Front. Immunol..

[112] de Mattos Grosso D., Almeida S.R., Mariano M., Lopes J.D. (2003). Characterization of gp70 and anti-gp70 monoclonal antibodies in *Paracoccidioides brasiliensis* pathogenesis. Infect. Immun..

[113] Buissa-Filho R., Puccia R., Marques A.F., Pinto F.A., Muñoz J.E., Nosanchuk J.D., Travassos L.R., Taborda C.P. (2008). The monoclonal antibody against the major diagnostic antigen of *Paracoccidioides brasiliensis* mediates immune protection in infected BALB/c mice challenged intratracheally with the fungus. Infect. Immun..

[114] Taborda C.P., Juliano M.A., Puccia R., Franco M., Travassos L.R. (1998). Mapping of the T-cell epitope in the major 43-kilodalton glycoprotein of *Paracoccidioides* brasiliensis which induces a Th-1 response protective against fungal infection in BALB/c mice. Infect. Immun..

[115] Toledo M.S., Tagliari L., Suzuki E., Silva C.M., Straus A.H., Takahashi H.K. (2010). Effect of anti-glycosphingolipid monoclonal antibodies in pathogenic fungal growth and differentiation. Characterization of monoclonal antibody MEST-3 directed to Man*p* α1→ 3Man*p* α1→ 2IPC. BMC Microbiol..

[116] Xander P., Vigna A., Feitosa L., Pugliese L., Bailão A., Soares C., Mortara R., Mariano M., Lopes J.A. (2007). Surface 75-KDa protein with acid phosphatase activity recognized by monoclonal antibodies that inhibit *Paracoccidioides* brasiliensis growth. Microbes Infect..

[117] Thomaz L., Nosanchuk J.D., Rossi D.C., Travassos L.R., Taborda C.P. (2014). Monoclonal antibodies to heat shock Monoclonal antibodies to heat shock protein 60 induce a protective immune response against experimental *Paracoccidioides lutzii*. Microb. Infect..

[118] Batista V.G., Toledo M.S., Straus A.H., Mendes-Giannini M.J., Duarte A.J., Takahashi H.K., Benard G. (2014). Glycolipid sensing and innate immunity in paracoccidioidomycosis. Mycopathologia.

[119] Bueno R.A., Thomaz L., Munoz J.E., da Silva C.J., Nosanchuk J.D., Pinto M.R., Travassos L.R., Taborda C.P. (2016). Antibodies against glycolipids enhance antifungal activity of macrophages and reduce fungal burden after infection with *Paracoccidioides brasiliensis*. Front. Microbiol..

[120] Shen H., Yu Y., Chen S.M., Sun J.J., Fang W., Guo S.Y., Hou W.T., Qiu X.R., Zhang Y., Chen Y.L. (2020). Dectin-1 Facilitates IL-18 Production for the Generation of Protective Antibodies against Candida albicans. Front. Microbiol..

[121] McEwen J.G., Sugar A.M., Brummer E., Restrepo A., Stevens D.A. (1984). Toxic effects of products of oxidative metabolism on the yeast form of *P. brasiliensis*. J. Med. Microbiol..

[122] Brummer E., Hanson L.H., Stevens D.A. (1988). Gamma-nterferon activation of macrophages for killing of *Paracoccidioides brasiliensis* and evidence for nonoxidative mechanisms. Int. J. Immunopharmacol..

[123] Nascimento F.R., Calich V.L.G., Rodriguez D., Russo M. (2002). Dual role for nitric oxide in paracoccidioidomycosis: Essential for resistance, but overproduction associated with susceptibility. J. Immunol..

[124] Nishikaku A.S., Molina R.F., Ribeiro L.C., Scavone R., Able B.P., Cunha C.S., Burger E. (2009). Nitric oxide participation in granulomatous response induced by *Paracoccidioides* brasiliensis infection in mice. Med. Microbiol. Immunol..

[125] Bernardino S., Pina A., Felonato M., Costa T.A., de Araújo E.F., Feriotti C., Bazan S.B., Keller A.C., Leite K.R.M., Calich V.L.G. (2013). TNF-α and CD8^+^ T cells mediate the beneficial effects of nitric oxide synthase-2 deficiency in pulmonary paracoccidioidomycosis. PLoS Negl. Trop. Dis..

[126] Livonesi M.C., Rossi M.A., de Souto J.T., Campanelli A.P., de Sousa R.L., Maffei C.M., Ferreira B.R., Martinez R., da Silva J.S. (2009). Inducible nitric oxide synthase-deficient mice show exacerbated inflammatory process and high production of both Th1 and Th2 cytokines during paracoccidioidomycosis. Microbes Infect..

[127] Lenzi H.L., Calich V.L.G., Miyaji M., Sano A., Nishimura K., Burger E. (1994). Fibrosis patterns of lesions developed by athymic and euthymic mice infected with *Paracoccidioides brasiliensis*. Braz. J. Med. Biol. Res..

[128] Burger E., Miyaji M., Sano A., Calich V.L.G., Nishimura K., Lenzi H.L. (1996). Histopathology of paracoccidioidomycotic infection in athymic and euthymic mice: A sequential study. Am. J. Trop. Med. Hyg..

[129] Souto J.T., Figueiredo F., Furlanetto A., Pfeffer K., Rossi M.A., Silva J.S. (2000). Interferon-γ and tumor Interferon-γ and Tumor Necrosis Factor-α determine resistance to *Paracoccidioides brasiliensis* infection in mice. Am. J. Pathol..

[130] Nishikaku A.S., Molina R.F.S., Albe B.P., Cunha C.S., Scavone R., Pizzo C.R.P., Camargo Z.P., Burger E. (2011). Immunolocalization of IFN-gamma in the lesions of resistant and susceptible mice to *Paracoccidioides brasiliensis* infection. FEMS Immunol. Med. Microbiol..

[131] Nishikaku A.S., Burger E. (2003). Immunohistochemical demonstration of TGF-ß and decorin in paracoccidioidal granulomas. Braz. J. Med. Biol. Res..

[132] Nascimento M.-P.P., Bannwart C.F., Nakaira-Takahagi E., Peraçoli M.T.S. (2011). Granulocyte macrophage colony-stimulating factor enhances the modulatory effect of cytokines on monocyte-derived multinucleated giant cell formation and fungicidal activity against *Paracoccidioides Bras*. Mem Inst. Oswaldo. Cruz..

[133] Tristão F.S.M., Rocha F.A., Carlos D., Ketelut-Carneiro N., Souza C.O.S., Milanezi C.M., Silva J.S. (2017). Th17-inducing cytokines IL-6 and IL-23 are crucial for granuloma formation during experimental paracoccidioidomycosis. Front. Immunol..

